# Pairing N‐Vacancy and Adjacent Ni‐Sites in the Local Microenvironment to Regulate the Urea Oxidation Reaction Pathway With Enhanced Kinetics

**DOI:** 10.1002/adma.202503879

**Published:** 2025-04-16

**Authors:** Chengwei Ji, Huimei Duan, Chuanhui Wang, Guizeng Liang, Xiaojing Long, Xilin She, Rongrong Zhang, Feilong Gong, Daohao Li, Dongjiang Yang, Jian Liu

**Affiliations:** ^1^ State Key Laboratory of Bio‐fibers and Eco‐textiles School of Environment and Geography College of Materials Science and Engineering Qingdao University Qingdao 266071 P. R. China; ^2^ State Key Laboratory of Inorganic Synthesis and Preparative Chemistry College of Chemistry Jilin University Changchun 130012 P. R. China; ^3^ Key Laboratory of Surface and Interface Science and Technology of Henan Province College of Material and Chemical Engineering Zhengzhou University of Light Industry Zhengzhou 450001 P. R. China; ^4^ Institute of Micro/Nano Materials and Devices Ningbo University of Technology Ningbo 315211 P. R. China; ^5^ Inner Mongolia Key Laboratory of Rare Earth Catalysis Science Center of Energy Material and Chemistry College of Chemistry and Chemical Engineering Inner Mongolia University Hohhot 010021 P. R. China; ^6^ DICP‐Surrey Joint Centre for Future Materials University of Surrey Guildford Surrey GU2 7XH UK

**Keywords:** charge redistribution, nitrogen vacancy, urea oxidation reaction, water electrolysis

## Abstract

The urea oxidation reaction (UOR) is a promising approach for replacing the oxygen evolution reaction in hydrogen production, offering lower energy consumption. However, the kinetics of Ni‐based catalysts for UOR are hindered by the high formation potential of NiOOH and its repeated transition with Ni(OH)_2_. In this study, a local microenvironment featuring electron‐deficient N‐vacancies (V_N_) paired with adjacent electron‐rich Ni‐sites on Ni_3_N (Ni_3_N‐V_N_) to enhance UOR kinetics is constructed. The electron‐rich Ni‐sites significantly reduce the energy barrier for NiOOH formation and promote the conversion of Ni(OH)_2_ to NiOOH. Meanwhile, the V_N_ sites induce low charge transfer resistance in Ni_3_N, facilitating efficient electron transfer and boosting UOR performance while ensuring the stability of the active NiOOH phase. The V_N_ sites promote the adsorption of the urea N atom at the active site, favoring the reaction pathway toward “NCO⁻” formation without requiring complete urea dissociation. This pathway alleviates the NiOOH/Ni(OH)_2_ conversion cycle, lowers charge transfer resistance, and improves reaction kinetics. Ni_3_N‐V_N_ demonstrates excellent UOR activity (low potential of 1.46 V at 1000 mA cm^−2^) and industrial prospects (integrating into an anion exchange membrane flow electrolyzer with 20% Pt/C, producing 600 mA cm^−2^ at 1.84 V), highlighting its potential for practical applications.

## Graphical abstract

In nitrogen vacancies‐rich nickel nitride (Ni_3_N‐V_N_), a local microenvironment of nitrogen vacancies and adjacent Ni sites is formed. The electron‐rich Ni‐sites can be converted to NiOOH at a lower potential, and urea adsorption on the electron‐deficient nitrogen vacancy region is conducive to its partial dissociation, conceiving to efficient urea oxidation reaction.

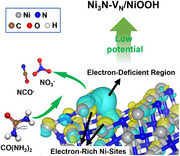

## 1 Introduction

The paradox of increasing energy demand and decreasing fossil fuel urges humankind to search for technologies to develop sustainable and clean energy sources.^[^ ^1^ ^]^ The use of water electrolysis to obtain green hydrogen has been considered the most promising hydrogen energy technology.^[^ ^2^ ^]^ However, the anode half‐reaction (oxygen evolution reaction, OER) of water splitting suffers from the intrinsically sluggish kinetics and a large thermodynamic potential of 1.23 V.^[^ ^3^ ^]^ These problems result in the high energy consumption of hydrogen production from water electrolysis. Using the thermodynamically advantageous urea oxidation reaction (UOR) with a small theoretical barrier (only 0.37 V) to replace the OER offers a promising strategy to reduce the energy consumption of hydrogen production from water electrolysis.^[^ ^4^, ^5^ ^]^ Hence, it is of urgent significance to pursue efficient UOR catalysts.

Nickel (Ni)‐based materials such as nickel oxide,^[^ ^6^ ^]^ nickel hydroxide,^[^ ^7^ ^]^ nickel sulfide,^[^ ^8^ ^]^ nickel phosphide,^[^ ^9^ ^]^ and nickel nitride,^[^ ^10^ ^]^ etc. are identified as excellent UOR catalysts due to their ability to dissociate C─N bonds of urea. In most cases, the basic principle of Ni‐based catalysts for UOR is believed to the indirect oxidation mechanism proposed by Botte et al. in 2013,^[^ ^11^ ^]^ the mechanism mentioned above can be represented as follows:

(1) $$6\mathrm{Ni}{\left(\mathrm{OH}\right)}_{2}+6OH^{-}\to 6\mathrm{NiOOH}+6H_{2}O+6e^{-}$$

(2) $$6\mathrm{NiOOH}+\mathrm{CO}{\left(NH_{2}\right)}_{2}+H_{2}O\to 6\mathrm{Ni}{\left(\mathrm{OH}\right)}_{2}+N_{2}+CO_{2}$$

In this reaction pathway, the surface of Ni‐based catalyst is oxidized to Ni^3+^ state (NiOOH), which serves as the active catalysis site for UOR. Then the urea is adsorbed on NiOOH through C or O atom and transforms into CO_2_ and N_2_ with 6e^−^ transfer, along with the reduction of NiOOH back to Ni(OH)_2_.^[^ ^12^ ^]^ Actually, complete dissociation of urea to CO_2_ and N_2_ is not ideal for boosting UOR kinetics, since the electron transfer of UOR depends on the conversion of Ni(OH)_2_ to NiOOH back and forth. The 6e^−^ conversions of Ni(OH)_2_/NiOOH in UOR require to overcome a high energy barrier, leading to a large charge transfer resistance (R_ct_), which impacts the reaction rate and overpotential by influencing the reaction kinetics.^[^ ^13^, ^14^, ^15^ ^]^ Therefore, the kinetics of UOR is significantly determined by the generation and regeneration of NiOOH species, particularly the regeneration process. Conversely, partial dissociation of urea such as NCO^−^, does not require as many electrons to participate in the reaction as complete dissociation species do.^[^ ^16^ ^]^ This greatly alleviates the reduction of NiOOH and thus leads to a lower R_ct_ and fast kinetics.

The establishment of urea adsorption configurations plays a key role in the control of UOR intermediate products, that is, the number of electron transfers. For instance, in most cases, the adsorption of C/O atom requires the transfer of 6e^−^ for one Ni‐site (complete dissociation: formation of CO_2_ and N_2_). However, the adsorption of N atom of urea is beneficial for partial dissociation: the generation of NCO^−^ and *NH with 1e^−^ transfer, while the oxidation of *NH at the active site to the final product NO_2_^−^ also requires only 4e^−^ transfer.^[^ ^16^, ^17^, ^18^ ^]^ This means that the N adsorption configuration is more conducive to reducing electron transfer, thereby reducing the reduction of NiOOH, which in turn lowers the impact of high R_ct_ caused by NiOOH reduction on the kinetics. According to the principles of thermodynamic adsorption, surface electron‐deficient region of the catalyst tends to adsorb the electron‐donating group (‐NH_2_) of urea, making the N atom of urea readily bind to the active sites to optimize UOR reaction pathway.^[^ ^5^, ^18^, ^19^ ^]^ Additionally, the initial generation of active NiOOH is seriously affected by the coordination environment of Ni‐sites,^[^ ^20^, ^21^ ^]^ the Ni‐sites with lower oxidation state have a relatively large number of electrons in the d orbital, and these electrons are relatively easy to lose, which makes it easier for the Ni sites to oxidize to high oxidation state NiOOH.^[^ ^21^, ^22^ ^]^ Therefore, an ideal Ni‐based UOR electrocatalyst should have both electron‐deficient regions and electron‐rich Ni sites to favor the generation and regeneration of active NiOOH species.

Herein, we introduced nitrogen‐vacancy (V_N_) into Ni_3_N grown on nickel foam (without or with V_N_ are denoted as Ni_3_N/NF and Ni_3_N‐V_N_/NF, respectively) to modulate the local microenvironment of V_N_ and adjacent Ni‐site. Density functional theory (DFT) calculations and experimental characterization proved that the electron‐rich state of Ni sites lowers the energy barrier for their transition to NiOOH and reduces the formation potential of NiOOH. At the same time, V_N_ enables Ni_3_N‐V_N_/NF to have a small charge transfer impedance, ensuring the charge transfer for boosting UOR, while ensuring the stability of active NiOOH. Moreover, the electron‐deficient V_N_ enhances the adsorption of an N atom of urea on the catalyst, which was conducive to the occurrence of incomplete urea dissociation into NCO^−^, alleviates the reduction of NiOOH, and thus reduces the charge transfer resistance, and enables Ni_3_N‐V_N_/NF exhibits faster reaction kinetics and superior UOR performance. The optimal Ni_3_N‐V_N_/NF shows exceptional UOR activity (a potential of just 1.46 V at a current density of 1000 mA cm^−2^), significantly lower than that of Ni_3_N/NF (1.60 V). The anion exchange membrane (AEM) flow electrolyzer combined with Ni_3_N‐V_N_/NF(M) anode and 20% commercial Pt/C cathode required only 1.84 V to achieve 600 mA cm^−2^.

## 2 Result and Discussion

### 2.1 Prediction of UOR Mechanism via DFT Calculations

**Figure 1:**
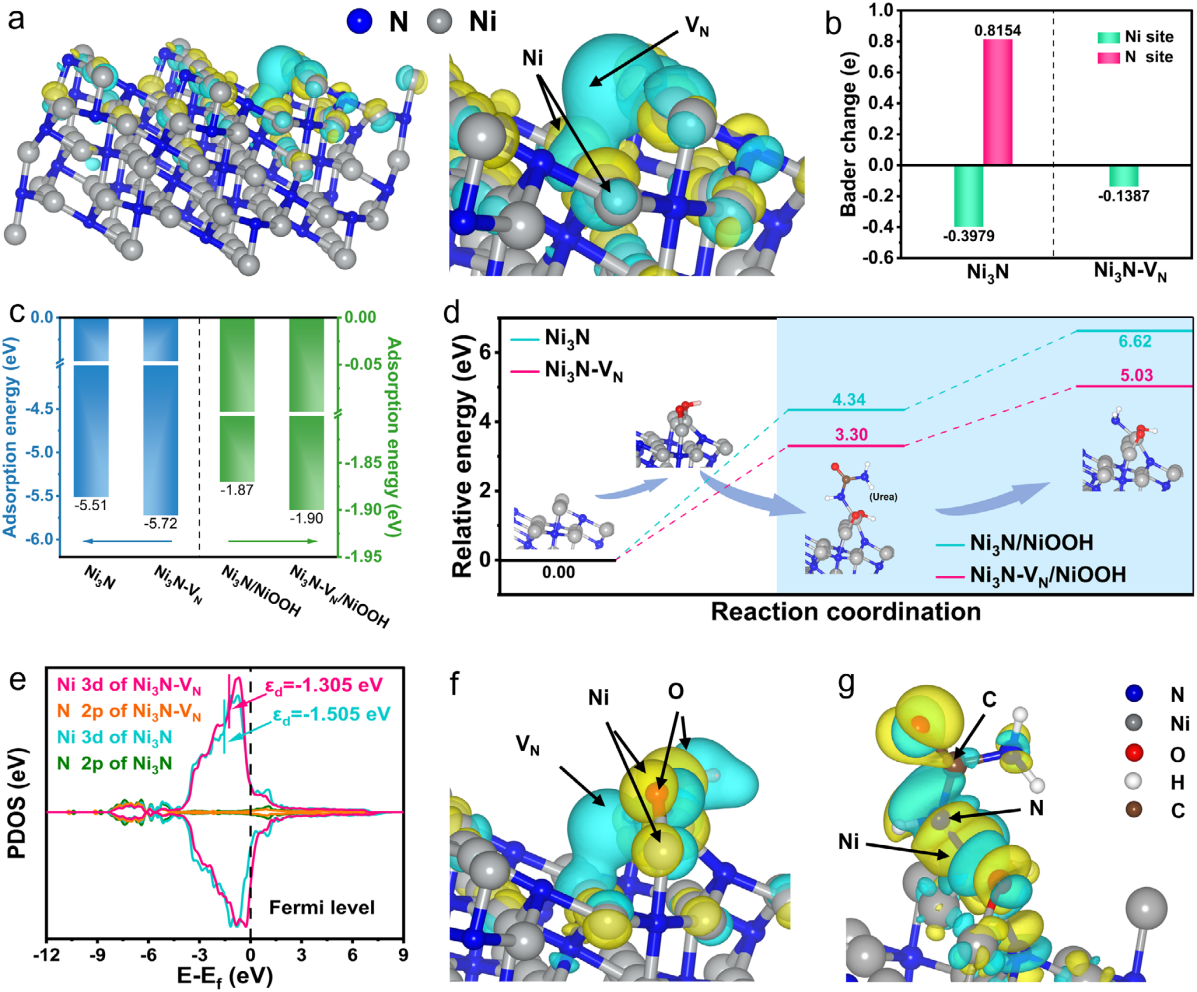
DFT Calculations of the UOR on pairing of N‐Vacancy and adjacent Ni‐sites local microenvironment. a) Change in charge density of Ni_3_N‐V_N_ for Ni_3_N. b) The Bader charge analysis diagram of Ni_3_N and Ni_3_N‐V_N_. c) The adsorption energy of urea on Ni_3_N, Ni_3_N‐V_N_, Ni_3_N/NiOOH and Ni_3_N‐V_N_/NiOOH. d) Free energy diagrams of intermediates in key steps of the UOR process on the catalyst surface. e) PDOS plots of Ni 3d band and N 2p band for Ni_3_N and Ni_3_N‐V_N_. f) Change in charge density of Ni_3_N‐V_N_/NiOOH for Ni_3_N/NiOOH. g) The charge density difference of urea molecule on Ni_3_N‐V_N_/NiOOH. For (a) and (f,g), the positive and negative charges are shown in yellow and cyan.

To predict the effect of the local microenvironment of V_N_ and adjacent Ni sites with the introduction of V_N_ on the UOR, DFT calculations were performed. Firstly, Models of Ni_3_N with and without V_N_ (Ni_3_N‐V_N_ and Ni_3_N) were constructed (Figure S1, Supporting Information) for study the change of the electronic state of Ni_3_N after introducing V_N_. Since V_N_ is formed by the loss of N atoms from Ni_3_N, it is widely distributed on the catalyst surface and all V_N_ sites are identical, the Ni_3_N‐V_N_ model is constructed by randomly replacing N atoms in Ni_3_N with V_N_. Charge density calculations show that V_N_ changes the electronic state of the Ni_3_N‐V_N_, creating the local microenvironment of V_N_ and adjacent Ni sites (**Figure** **1**a). The Bader charge analysis (Figure 1b) reveals the electron transfer propensity, the average charge of Ni atoms near the V_N_ in Ni_3_N‐V_N_ is −0.1387 |e|, which is much lower than the average charge of Ni atoms in the same positions in Ni_3_N (−0.3979 |e|). This indicates that the Ni atoms near V_N_ in Ni_3_N‐V_N_ are electron‐rich compared to those in Ni_3_N. This further confirms that introducing V_N_ causes electron delocalization at the vacancy center to form an electron‐deficient regions. Concurrently, it reduces the electron removal from Ni atoms near V_N_, creating electron‐rich regions at the Ni sites. Second, the adsorption of urea molecules on Ni_3_N‐V_N_ (or Ni_3_N‐V_N_/NiOOH) and Ni_3_N (or Ni_3_N/NiOOH) was discussed. The adsorption of N atom on the catalyst is stronger than that of C/O atom, suggesting that urea tends to be adsorbed on the catalyst by N atom. The slightly stronger adsorption of urea molecules on Ni_3_N‐V_N_ (or Ni_3_N‐V_N_/NiOOH) compared to Ni_3_N (or Ni_3_N/NiOOH) indicates that electron‐deficient V_N_ optimize urea adsorption (Figures S2 and S3, Supporting Information; and Figure 1c). Thirdly, the process of Ni sites transforming into NiOOH was discussed. Gibbs free energy calculations revealed that compared to Ni_3_N (4.34 eV), Ni_3_N‐V_N_ (3.30 eV) has a lower energy barrier for NiOOH generation (Figure 1d).

The partial density of states (PDOS) analysis showed that compared to Ni_3_N, the d‐band center of Ni sites in Ni_3_N‐V_N_ is closer to the Fermi level, facilitating easier electron excitation to higher energy states favorable for NiOOH formation (Figure 1e).^[^ ^13^, ^23^ ^]^ This suggests that the introduction of V_N_ optimizes the local electron distribution, generates electron‐rich Ni sites, and promotes the transformation of Ni sites to NiOOH. In addition, after the generation of NiOOH on Ni_3_N‐V_N_, the local microenvironment of V_N_ and adjacent Ni sites remained in the original state (Figure S4, Supporting Information; and Figure 1f), which was conducive to the stability of UOR. Fourthly, the advantages of Ni_3_N‐V_N_ for the “NCO^−^” pathway were explored. By comparing the differential charges of Ni_3_N‐V_N_/NiOOH and Ni_3_N/NiOOH adsorbent urea, Differential charge diagrams clearly demonstrate significant electron exchange around the C─N bond of urea adsorbed on Ni_3_N‐V_N_/NiOOH (Figure S5, Supporting Information; and Figure 1g). Bader charge analysis (Figure S6, Supporting Information) provides more intuitive evidence: the charge on the N atom of the C─N bond adsorbed on Ni_3_N‐V_N_/NiOOH (1.14 |e|) is higher than that on Ni_3_N/NiOOH (1.07 |e|). The increased electronegativity disrupts the original charge transfer balance, facilitating C─N bond dissociation. The lower dissociation potential (1.73 eV) of the C─N bond on Ni_3_N‐V_N_ is also much lower than that of Ni_3_N (2.28 eV) (Figure 1d). This confirms the predominated “NCO^−^” pathway occurs on the Ni_3_N‐V_N_ catalysts. Finally, the stability of NCO^−^ in the system is analyzed. By comparing the dissociation energies of the C═N bond in NCO^−^ adsorbed on the catalyst (2.68 eV) with the C─N bond in urea adsorbed on the catalyst (1.73 eV), it was found that breaking the C═N bond requires a higher barrier than breaking the C─N bond (Figure S7, Supporting Information). Therefore, in the presence of urea, it is difficult for NCO^−^ to further oxidize at the active site, which ensures the stability of NCO^−^ in the UOR reaction system and ensures the excellent kinetics of UOR.

### 2.2 Synthesis and Characterization of Ni_3_N/NF and Ni_3_N‐V_N_/NF

**Figure 2:**
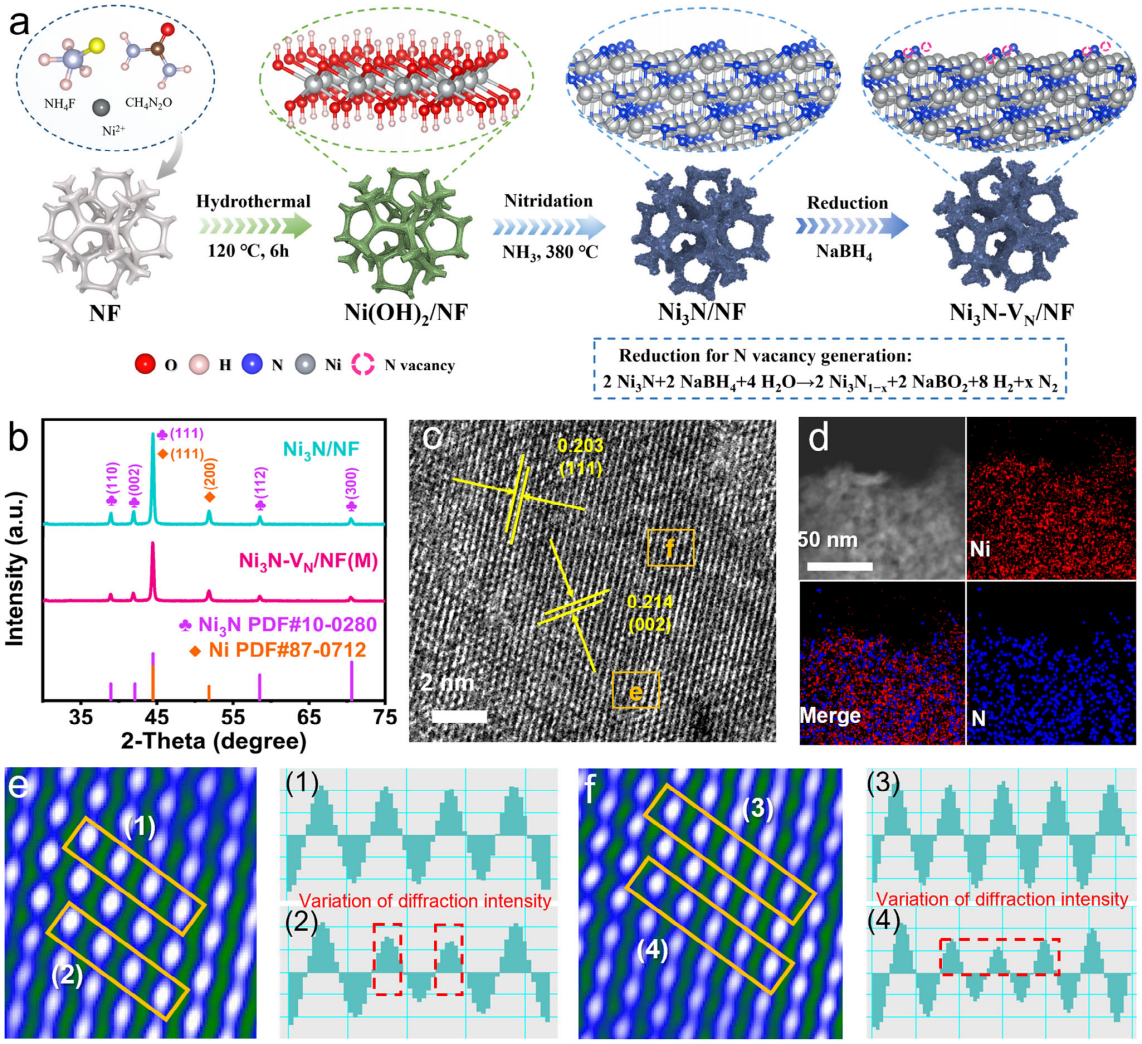
Preparation and characterization of Ni_3_N/NF and Ni_3_N‐V_N_/NF. a) Schematic of the preparation method of Ni_3_N‐V_N_/NF. b) XRD patterns of Ni_3_N/NF and Ni_3_N‐V_N_/NF(M). c) HRTEM image of Ni_3_N‐V_N_/NF(M). d) HAADF‐STEM and elemental mapping images of Ni and N elements for Ni_3_N‐V_N_/NF(M). e, f) Corresponding filtered images of the orange square in (c). 1–4) normalized intensity changes of the corresponding columns in (e) and (f).

Based on the above forecast, the Ni_3_N‐V_N_/NF catalyst was prepared, and the synthesis process was shown in **Figure** **2**a. First, Ni(NO_3_)_2_·6H_2_O, urea, and NH_4_F were dissolved in water and placed together with nickel foam in an autoclave at 120 °C for 6 h to obtain the Ni(OH)_2_ precursor grown on nickel foam (Ni(OH)_2_/NF). Subsequently, Ni(OH)_2_/NF was annealed in ammonia atmosphere at 380 °C to obtain Ni_3_N/NF. And then, NaBH_4_ was used as a reducing agent to reduce Ni_3_N. During the reaction, the H^−^ ions generated by the NaBH_4_ solution attack the Ni─N bonds, resulting in the removal of some N atoms from the lattice and introducing V_N_ into Ni_3_N. By controlling the reduction time, Ni_3_N‐V_N_/NF with low, medium, and high V_N_ density (Ni_3_N‐V_N_/NF(L), Ni_3_N‐V_N_/NF(M), and Ni_3_N‐V_N_/NF(H)) were obtained. The lower V_N_ density would not enhance the electrochemical performance sufficiently, and the too high V_N_ density would lead to vacancy aggregation, resulting in structural instability and affect the electron distribution and migration ability. Only when the V_N_ density is moderate, the number of active sites and the stability of the catalyst reach a balance point, thus showing the best catalytic performance.^[^ ^24^ ^]^ The crystal structures of the catalysts were performed by X‐ray diffraction (XRD). For all catalysts (Figure 2b; Figure S8, Supporting Information), the peaks at 38.9°, 42.1°, 44.5°, 58.5°, and 70.6° belong to the (110), (002), (111), (112), and (300) planes of Ni_3_N (JCPDS No. 10–0280),^[^ ^25^, ^26^, ^27^, ^28^, ^29^ ^]^ confirming that V_N_ does not affect the crystal structure of Ni_3_N. The scanning electron microscopy (SEM) image shows that the Ni_3_N‐V_N_/NF(M) displays a flower‐like porous structure composed of interconnected nanosheets (Figure S9d–f, Supporting Information), which inherits from the Ni(OH)_2_/NF and Ni_3_N/NF samples (Figures S9a–c; S10, Supporting Information). This structure ensures a large contact area between the electrolyte and the catalyst, facilitating electrolyte penetration and gas release. The powder samples (Ni_3_N‐V_N_) scraped from Ni_3_N‐V_N_/NF were used for transmission electron microscopy (TEM) and high‐resolution TEM (HRTEM) characterization. The TEM and HRTEM images exhibit the lattice spacings of 0.203 and 0.214 nm of Ni_3_N‐V_N_ (Figure S11, Supporting Information; and Figure 2c), corresponding to (111) and (002) planes of Ni_3_N (JCPDS NO. 10–0280).^[^ ^26^, ^29^, ^30^ ^]^ The selected area electron diffraction (SAED) pattern exhibits a poly‐crystalline structure of Ni_3_N‐V_N_, with (112), (111), (002), and (110) planes (Figure S12, Supporting Information). High‐angle annular dark field scanning TEM (HAADF‐STEM) and energy dispersive X‐ray elemental mapping unravel the uniform distribution of Ni and N elements in Ni_3_N‐V_N_ (Figure 2d). Figure 2e,f show the filtered image of orange square in Figure 2c and the normalized intensity variations of the tagged columns. In the normalized intensity images, the intensity distribution is proportional to the atomic weights passing through the corresponding atomic column along the beam distribution direction. The varied diffracted intensity clearly indicates the change in the atomic column distribution in Ni_3_N‐V_N_ (Figure 2e,f), possibly due to V_N_.^[^ ^31^ ^]^

**Figure 3:**
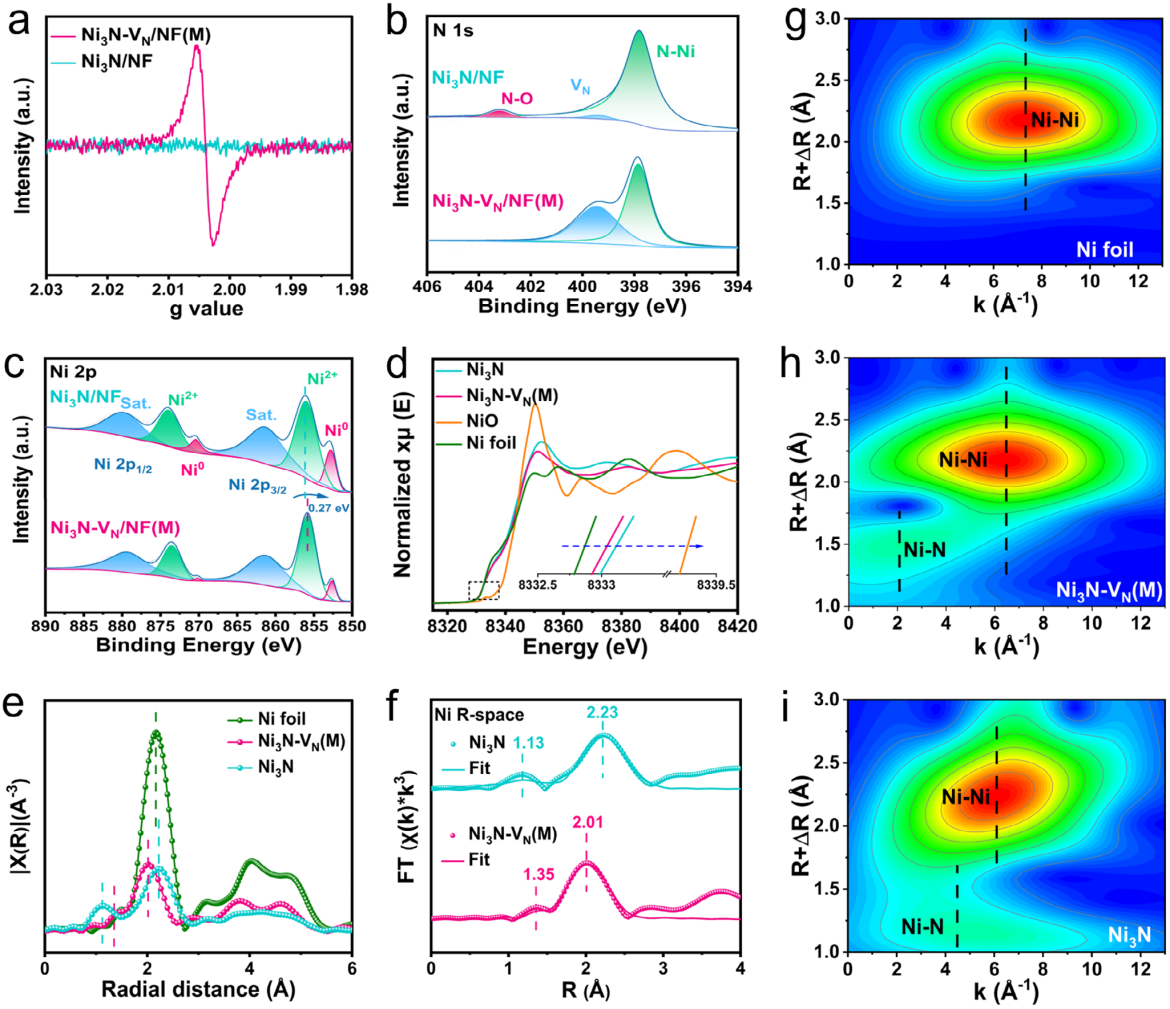
Electronic and structural characterization of the prepared electrocatalysts. a) EPR spectrum of Ni_3_N/NF and Ni_3_N‐V_N_/NF(M). XPS spectra of N 1s b) and Ni 2p c) for Ni_3_N/NF and Ni_3_N‐V_N_/NF(M). Ni K‐edge XANES d) and FT‐EXAFS e) for Ni_3_N, Ni_3_N‐V_N_(M), and Ni foil, with control samples of NiO also shown in d). f) The best‐fit EXAFS spectra in R space for Ni_3_N and Ni_3_N‐V_N_(M). g–i) WT‐EXAFS contour plots of the Ni foil, Ni_3_N‐V_N_(M), and Ni_3_N.

The electronic states of V_N_ and adjacent Ni sites in Ni_3_N‐V_N_ were studied. The V_N_ of Ni_3_N‐V_N_/NF was characterized by electron paramagnetic resonance (EPR). Compared with Ni_3_N/NF, Ni_3_N‐V_N_/NF has a distinct EPR signal (g value 2.004), and the signal strength increases with the increase of the reduction time (Figure S13, Supporting Information; and **Figure** **3**a), indicating that V_N_ is successfully introduced into Ni_3_N‐V_N_/NF. The surface charge states of catalysts were investigated by X‐ray photoelectron spectroscopy (XPS) (Figure S14, Supporting Information). The high‐resolution XPS spectrum of N 1s shows peaks for Ni─N bond and V_N_.^[^ ^27^, ^32^ ^]^ The surface V_N_ concentrations (based peak area ratio of XPS) of Ni_3_N/NF, Ni_3_N‐V_N_/NF(L), Ni_3_N‐V_N_/NF(M), and Ni_3_N‐V_N_/NF(H) are 1.8% (due to the reducibility of NH_3_), 29.7%, 42.5%, and 62.6%, respectively, which also supports the EPR results (Figure 3b; and Figure S14b,c, Supporting Information).^[^ ^32^ ^]^ For the Ni 2p high‐resolution XPS spectra of Ni_3_N/NF, peaks at 852.6 and 870.1 eV correspond to Ni^0^ from the nickel foam, while peaks at 855.2 and 873.4 eV correspond to 2p_3/2_ and 2p_1/2_ of Ni^2+^ (Figure 3c).^[^ ^28^, ^29^ ^]^ The Ni 2p signal of Ni_3_N‐V_N_/NF(M) is reduced by ≈0.27 eV compared with Ni_3_N/NF, indicating that Ni in Ni_3_N‐V_N_/NF(M) is electron‐rich relative to Ni in Ni_3_N/NF.^[^ ^28^, ^30^ ^]^ The electronic states of Ni_3_N‐V_N_/NF was further studied using X‐ray absorption fine structure (XAFS) spectroscopy. X‐ray absorption near edge structure (XANES) analyses of Ni_3_N and Ni_3_N‐V_N_(M) are compared with control samples of Ni foil and NiO (Figure 3d). To avoid the influence of NF, the powder samples (Ni_3_N and Ni_3_N‐V_N_(M)) were synthesized and characterized by the same method. The XANES shows that the Ni K‐edge absorption energies of Ni_3_N and Ni_3_N‐V_N_(M) are between those of Ni foil and NiO, suggesting the average valence state of Ni atoms in Ni_3_N and Ni_3_N‐V_N_(M) is between 0 and +2. Specifically, the valence state of Ni in Ni_3_N‐V_N_(M) is closer to 0, further indicating V_N_ makes the nearby Ni appear electron‐rich. The extended X‐ray absorption fine structure (EXAFS) spectrum of Ni_3_N exhibits peaks at 1.13 and 2.23 Å for Ni‐N and Ni‐Ni bonds (Figure 3e).^[^ ^29^, ^30^ ^]^ In Ni_3_N‐V_N_(M), the Ni‐N bond is located at 1.35 Å, which is 0.22 Å to the right of the Ni‐N bond in Ni_3_N. This indicates that the introduction of V_N_ alters the local coordination environment, affecting the interactions between atoms. As a result, Ni atoms tend to form stronger metallic bonds with neighboring Ni atoms, leading to the shortening of Ni‐Ni bonds. At the same time, the Ni‐N bonds are stretched, and the electron‐donating ability of Ni is weakened, which results in the formation of electron‐rich Ni sites in Ni_3_N‐V_N_(M). The EXAFS curve fitting results further clarify the above structural transformation, and the reduction in the coordination number of Ni‐N bonds also provides evidence for the successful incorporation of V_N_ (Figure 3f; and Table S1, Supporting Information).^[^ ^33^ ^]^In the wavelet transform of the EXAFS spectrum (Figure 3g–i) obtained from the K‐space data (Figure S15, Supporting Information), Ni_3_N displays signals at 4.7 and 6.2 Å^−1^ in the K‐space for Ni‐N and Ni‐Ni bonds, respectively. Ni_3_N‐V_N_(M) shows the Ni‐N bond signal and the Ni‐Ni bond signal shift to 2.1 Å^−1^ and 6.6 Å^−1^ in K‐space, respectively.^[^ ^29^ ^]^ This change further confirms that V_N_ modulates the electronic environment in Ni atom, which is consistent with DFT calculations. In other words, by introducing V_N_, the local microenvironment of V_N_ and adjacent Ni sites is adjusted to form a pairing of electron‐rich Ni sites and electron‐deficient V_N_.

### 2.3 Electrocatalytic UOR Performance

**Figure 4:**
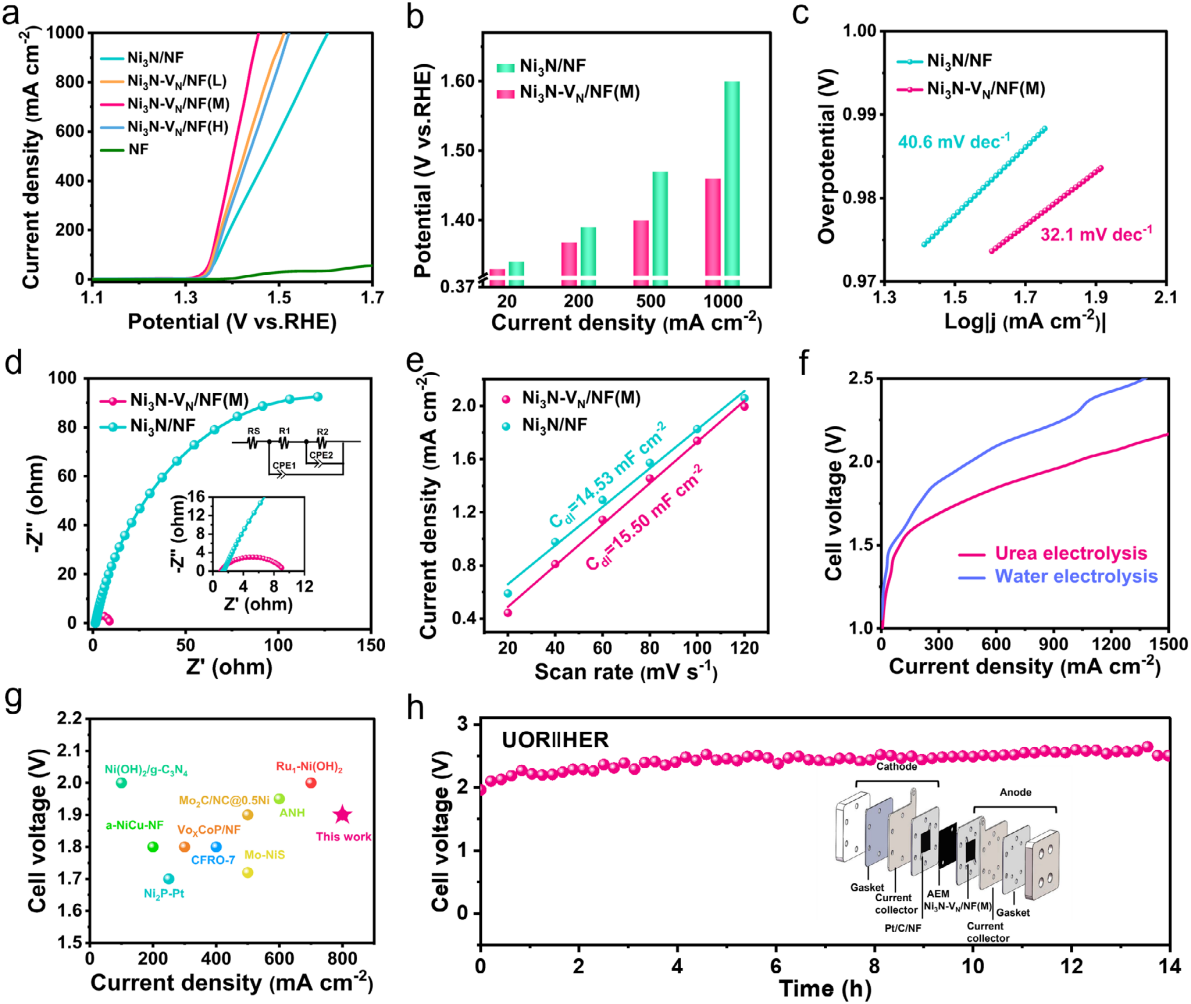
Electrocatalytic UOR performance of the prepared electrocatalysts. a) UOR LSV curves of all catalysts in 1.0 _M_ KOH + 0.33 _M_ urea. b) Potential of all catalysts in UOR. c) Tafel plots in 1.0 _M_ KOH + 0.33 _M_ urea. d) EIS Nyquist plot for UOR of Ni_3_N/NF and Ni_3_N‐V_N_/NF(M), the insets display the high‐frequency range of Nyquist plots and the equivalent circuit model, respectively. e) Double‐layer capacitance (C_dl_) plots. f) Polarization curve of Pt/C/NF (cathode) versus Ni_3_N‐V_N_/NF(M) (anode) for water electrolysis and urea electrolysis. g) Comparison of Ni_3_N‐V_N_/NF(M)‖Pt/C/NF with previously reported AEM electrolyzers. h) Stability test of Pt/C/NF (cathode) versus Ni_3_N‐V_N_/NF(M) (anode), the insert displays a schematic illustration of the AEM electrolyzer test unit.

The OER and UOR performance of prepared catalysts were evaluated in a three‐electrode system. The linear sweep voltammetry (LSV) curves in 1 M KOH + 0.33 M urea showed Ni_3_N‐V_N_/NF(M) outperformed Ni_3_N‐V_N_/NF(L), Ni_3_N‐V_N_/NF(H), Ni_3_N/NF, nickel foam, and most reported Ni_3_N‐based catalysts (**Figure** **4**a; Table S2, Supporting Information). Specifically, Ni_3_N‐V_N_/NF(M) achieves current densities of 20/1000 mA cm^−2^ at low potentials of 1.33/1.46 V, outperforming Ni_3_N/NF (E_20/1000_ = 1.34/1.60 V) (Figure 4b). Notably, Ni_3_N/NF and Ni_3_N‐V_N_/NF(M) show poor OER activity (Figure S16a, Supporting Information). After repeated cyclic voltammetry (CV) cycles, the activity of Ni_3_N‐V_N_/NF(M) shows an increasing trend, while the activity of Ni_3_N/NF remains relatively stable. This might be related to the in situ transformation of active sites into NiOOH, followed by the consumption and accumulation of NiOOH (Figure S16b,c, Supporting Information). Tafel slopes (Figure 4c) derived from LSV and electrochemical impedance spectroscopy (EIS) (Figure 4d) were used further to investigate the UOR reaction kinetics of the catalysts. Ni_3_N‐V_N_/NF(M) shows a lower Tafel slope (32.1 mV dec⁻¹) compared to Ni_3_N/NF (40.6 mV dec⁻¹).

The Nyquist diagram displays a smaller semicircular radius than Ni_3_N/NF, indicating lower R_ct_. All these indicate that Ni_3_N‐V_N_/NF(M) enhances the reaction kinetics due to its unique electronic structure. Additionally, double‐layer capacitance (C_dl_) was calculated via CV to assess its intrinsic catalytic activity, which is highly correlated to the electrochemically active surface area (Figure S16d,e, Supporting Information). Ni_3_N‐V_N_/NF(M) exhibits a large C_dl_ value of 15.50 mF cm^−2^ compared to Ni_3_N/NF (14.53 mF cm^−2^), indicating a larger electrochemical surface area and more exposed active sites (Figure 4e). In addition, Ni_3_N‐V_N_/NF(M) maintains a current density of 100 mA cm^−2^ at a constant voltage in UOR for 50 h and still maintains the initial 87.72%, showing good stability (Figure S17, Supporting Information). Furthermore, its industrial feasibility was tested in an AEM electrolyzer with Ni_3_N‐V_N_/NF(M) anode and 20% commercial Pt/C cathode (Figure S18, Supporting Information; and Figure 4h). The catalyst achieves a current density of 600 mA cm^−2^ at 1.84 V with urea assistance (Figure 4f), which is better than water electrolysis without urea and exceeds the performance of other reported AEM electrolyzers for urea‐coupled hydrogen production (Figure 4g; and Table S3, Supporting Information). The system operates stably for 14 h at 2 V (constant current: 1 A cm^−2^), demonstrating good stability (Figure 4h). As an important factor showing the economic efficiency of the electrolyzer, the price per gasoline‐gallon equivalent H_2_ is as low as $ 0.96 according to the calculations in Supporting Information (Note S1, Supporting Information), much lower than the target of U.S. DOE by 2026 ($ 2.00).

### 2.4 UOR Mechanism Analysis

**Figure 5:**
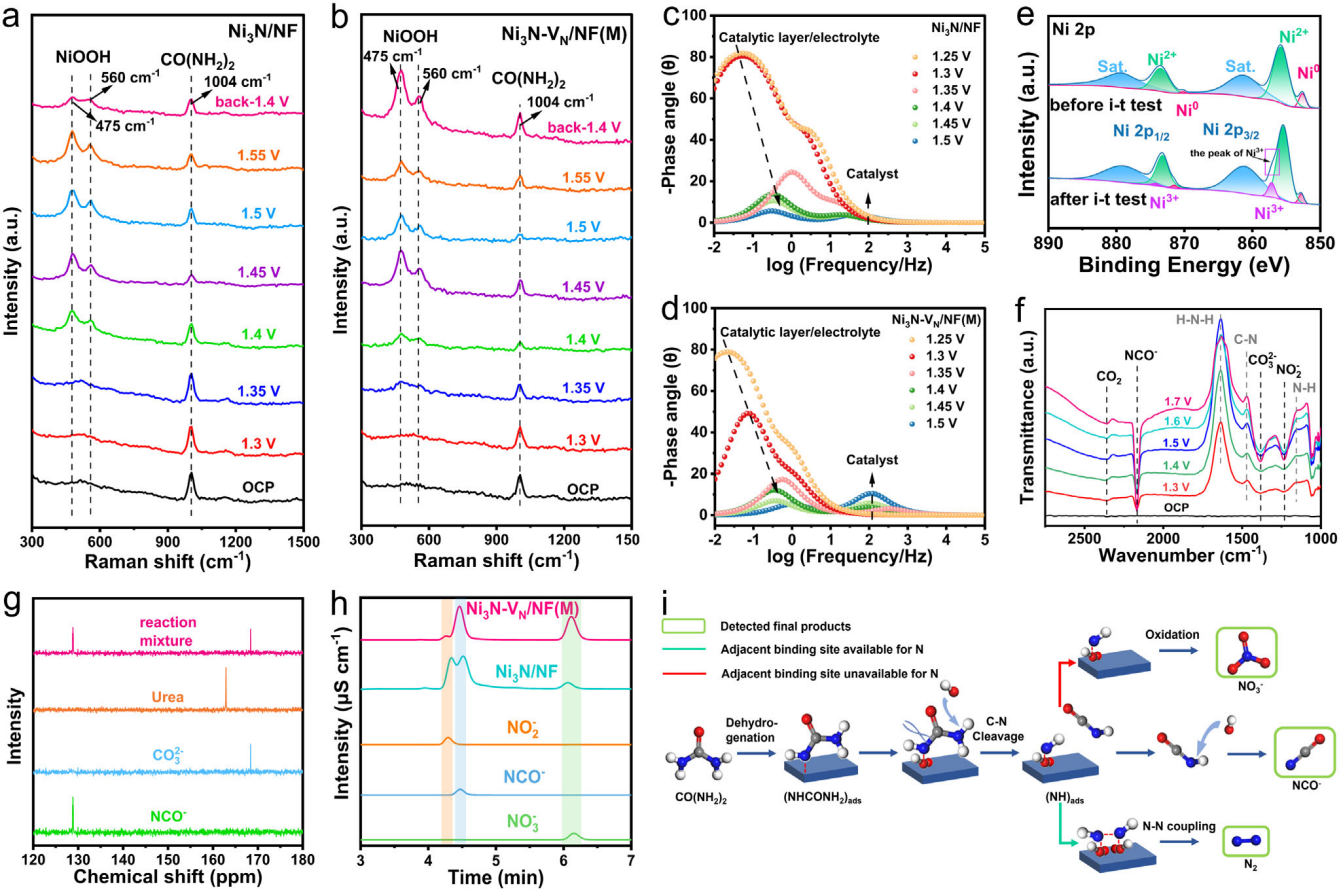
Mechanism of the UOR on the Ni_3_N‐V_N_/NF. The potential‐resolved in situ Raman spectra of Ni_3_N/NF a) and Ni_3_N‐V_N_/NF(M) (b) in 1.0 _M_ KOH + 0.33 _M_ urea. Potential‐dependent EIS Bode plots of Ni_3_N/NF c) and Ni_3_N‐V_N_/NF(M) d) when potentials varied from 1.25 to 1.5 V. e) XPS Ni 2p comparison of the initial and recovered Ni_3_N‐V_N_/NF(M). f) In situ FTIR spectra obtained of Ni_3_N‐V_N_/NF(M). g) An example of the NMR spectra recorded for long‐term UOR at the Ni_3_N‐V_N_/NF(M) anode compared to the NMR spectra of several carbon‐containing anions and urea (2.0 V vs RHE, without IR compensation). h) Examples of the IC traces recorded for UOR reaction mixture of Ni_3_N/NF and Ni_3_N‐V_N_/NF(M) at 2.0 V (vs RHE, without IR compensation) compared to several standard anion solutions. i) A schematic illustration of the proposed pathways for UOR on Ni_3_N‐V_N_ surface.

To determine the roles of the the local microenvironments of V_N_ and adjacent Ni sites in the UOR process and confirm the prediction of DFT, we performed a series of characterizations, starting with the potential‐resolved in situ Raman tests were performed to reveal the true active site in the structure evolution of the Ni_3_N‐V_N_/NF catalysts during UOR process. As shown in **Figure** **5**a,b, at open circuit potential (OCP) and initial potential, the Raman spectra of both catalysts show only the urea peak (1004 cm^−1^) due to the adsorption of urea onto the catalyst surface.^[^ ^34^, ^35^ ^]^ When the applied potential increases to 1.35 V (vs RHE), Ni_3_N‐V_N_/NF(M) firstly exhibits peaks of NiOOH (475 and 560 cm^−1^), which is ascribed to the reconfiguration of the surface Ni‐sites into NiOOH species.^[^ ^34^, ^35^, ^36^ ^]^ Furthermore, Ni^3+^ (857.2 and 874.8 eV) appeared in the XPS spectra of Ni_3_N‐V_N_/NF(M) after the reaction (Figure 5e) also supports the formation of NiOOH.^[^ ^29^ ^]^ For Ni_3_N/NF, it takes a higher voltage of 1.4 V to form the NiOOH species (Figure 5b). The time‐resolved in situ Raman is also performed at 1.35 V to confirm the stability of NiOOH, which is generated by Ni_3_N‐V_N_ reconstruction, and Ni_3_N never reconstructs to NiOOH at 1.35 V (Figure S19, Supporting Information). This means that electron‐rich Ni sites can promote the conversion of Ni species to NiOOH species, because the lower state of Ni has a more suitable d‐band center to promote the formation of NiOOH.^[^ ^13^ ^]^ With the voltage returning to 1.4 V, the NiOOH peaks in Ni_3_N‐V_N_/NF(M) gradually accumulate, indicating that indirect oxidation occurred and more NiOOH is generated than consumed. However, when the voltage returns to 1.4 V, the Raman peak of NiOOH of Ni_3_N/NF is not significant. Apparently, the electron‐deficient V_N_ are crucial to stabilizing the formed NiOOH species. The same conclusion is also drawn from the EIS, where the EIS data of Ni_3_N‐V_N_/NF(M) and Ni_3_N/NF were recorded at 1.3 V, 1.5 V, and back to 1.3 V to observe the changes in R_ct_. For Ni_3_N/NF, during the process from 1.3 to 1.5 V and back to 1.3 V, the R_ct_ first decreases and then increases. For Ni_3_N‐V_N_/NF(M), the R_ct_ decreases and then remains unchanged. This is consistent with the in situ Raman results, reflecting the process where Ni_3_N/NF generates NiOOH during the indirect oxidation reaction, and NiOOH is subsequently consumed, while for Ni_3_N‐V_N_/NF(M), NiOOH is generated and remains stable (Figure S20, Supporting Information). In situ Raman data for Ni_3_N‐V_N_/NF(M) in 1 _M_ KOH were also recorded (Figure S21, Supporting Information). In 1 _M_ KOH, NiOOH appears at 1.4 V and cannot remain stable, demonstrating the superior performance of Ni_3_N‐V_N_/NF(M) in UOR. In addition, the reconstruction of NiOOH and electron transfer of the catalysts during UOR were further studied by recording the EIS at different potentials. The Bode plots describe two electrochemical processes on the Ni_3_N/NF and Ni_3_N‐V_N_/NF(M) electrodes (Figure 5c,d), showing the phase angle in the high‐frequency region for internal resistance of the electrode, and the phase angle in the low‐frequency region for electron transfer from the electrolyte to the catalytic layer. For Ni_3_N‐V_N_/NF(M), the appearance of phase angle in the high‐frequency region at above 1.35 V is derived from the abundant low‐conductivity NiOOH, which indicates the more stability of the true NiOOH active site in Ni_3_N‐V_N_ than that of Ni_3_N.^[^ ^37^ ^]^ Simultaneously, the phase angle of Ni_3_N‐V_N_/NF(M) in the low‐frequency region between 1.25–1.35 V decreases faster than that of Ni_3_N/NF, resulting in the generation of NiOOH at low potential for Ni_3_N‐V_N_, which consists with the Raman results. Moreover, the smaller phase angle in the low‐frequency of Ni_3_N‐V_N_ compared with Ni_3_N indicates the fast charge transfer, which benefits the UOR kinetics.^[^ ^37^, ^38^ ^]^ These results strongly demonstrate that the phase transition energy barrier and electron transfer barrier for Ni_3_N‐V_N_/NF(M) are lower than those for Ni_3_N/NF, resulting in faster reaction kinetics. Overall, the pairing of electron‐deficient V_N_ and electron‐rich Ni sites local microenvironments reduce the formation potential of NiOOH and guarantee fewer NiOOH/Ni(OH)_2_ conversion, thereby synergistically optimizing the UOR overpotential and kinetics of Ni_3_N‐V_N_/NF(M). The stability of catalyst structure is the key to its electrochemical stability. Ni_3_N‐V_N_/NF(M) was characterized after 50 h i‐t test. XPS (Figure S22, Supporting Information; and Figure 5e) show the presence of Ni^3+^, indicating the formation of NiOOH. The main phase of the catalyst is Ni_3_N after the i‐t test (Figure S23a, Supporting Information). In addition, the EPR (Figure S23b, Supporting Information) spectra of Ni_3_N‐V_N_/NF(M) showed no significant changes, which was consistent with the conclusion of DFT, indicating the local microenvironments of V_N_ and Ni sites remain on the Ni_3_N‐V_N_/NF(M) catalyst after UOR.

Then we analyzed the UOR products and first detected the intermediates and liquid‐phase products of UOR on Ni_3_N/NF and Ni_3_N‐V_N_/NF(M). The reaction intermediates of Ni_3_N‐V_N_/NF(M) at different potentials were investigated using in situ FTIR spectroscopy (Figure 5f).^[^ ^15^ ^]^ Compared with the FTIR spectra at OCP, higher potentials show positive peaks at ca. 1630 (δ(HNH) vibrations), 1460 (ν(CN) vibration), and 1150 cm^−1^ (ρ(NH_2_) vibration), which is assigned to urea consumption. The strongest δ(HNH) vibration peak indicates that the urea is adsorbed on the catalyst by N atoms. As the intensity of the urea consumption peak increases, three negative‐pointing absorption peaks appear at ca. 1230, 1380, 2160, and 2360 cm^−1^, which can be attributed to the formation of NO_2_^−^, CO_3_^2−^, NCO^−^, and CO_2_, respectively. For Ni_3_N/NF, the intensity of the peak corresponding to NCO^−^ is relatively weak (Figure S24, Supporting Information), indicating that the proportion of NCO^−^ in the UOR product of Ni_3_N/NF is lower than that of Ni_3_N‐V_N_/NF(M). Chronoamperometric tests were conducted on Ni_3_N/NF and Ni_3_N‐V_N_/NF(M) at 1.5 and 2.0 V (vs RHE). After a significant decrease in current, the electrolytes were collected and analyzed using ion chromatography and ^13^C nuclear magnetic resonance (NMR) spectroscopy, at which point urea was almost completely consumed in both electrolytes. The data reveal that the primary UOR products of Ni_3_N/NF and Ni_3_N‐V_N_/NF(M) are NO_x_^−^, NCO^−^, NH_4_^+,^ and CO_3_^2−^ at both low and high voltages (Figure 5g,h; Figure S25, Supporting Information; and Table S4, Supporting Information). Considering the results obtained here and prior literature,^[^ ^35^, ^39^ ^]^ we proposed a combined “NCO^−^” and “NH_3_ + CO_2_” UOR reaction pathway of Ni_3_N‐V_N_ and Ni_3_N. Specifically, urea is firstly adsorbed on the surfaces of Ni_3_N‐V_N_ and Ni_3_N through the N atom of one ─NH_2_ group at the OCP. As the reaction progresses, the Ni sites on the catalyst surface gradually transform into NiOOH at higher potentials. In situ Raman spectroscopy (Figure S21, Supporting Information) confirms that NiOOH forms in 1 _M_ KOH even in the absence of urea. However, the presence of urea stabilizes the NiOOH phase by modulating the local microenvironment, which enhances the UOR kinetics. In the “NH_3_ + CO_2_” pathway (Figure S26, Supporting Information), the C─N bond adjacent to the unadsorbed ‐NH_2_ group of urea (the distal C─N bond) was first broken and converted to NH_4_^+^. Subsequently, the adsorbed C─N bond (the proximal C─N bond) dissociates to release CO_2_ and thus to form CO_3_^2−^ in solution. However, if the proximal C─N bond (adjacent to the adsorbed ‐NH_2_ group of urea) breaks first, a very different “NCO^−^” pathway occurs (Figure 5i; Figure S27, Supporting Information). In other words, the proximal C─N bond decomposes into NCO^−^, while the distal C─N bond of the NCO^−^ species could not be further catalyzed to decompose, and the retained NCO^−^ can be used as a precursor to produce isocyanate. In both the “NCO^−^” and “NH_3_ + CO_2_” pathways, any remaining *NH adsorbed on the surface is further oxidized to NO_x_^−^ (if the adjacent binding site is unavailable for N) or undergoes N‐N coupling to form N_2_ (if the adjacent binding site is available for N). It should be noted that for the UOR process involving Ni_3_N/NF, the nitrogen content in NH_4_^+^ in the product is similar to that of NCO^−^, indicating that the “NCO^−^” pathway holds an equivalent status to the “NH_3_+CO_2_” pathway. However, for the UOR process in which Ni_3_N‐V_N_/NF(M) participated, the proportion of NCO^−^ increases significantly compared to NH_4_^+^ (Table S4, Supporting Information), suggesting that the “NCO^−^” pathway predominates. This is due to that the local microenvironment of V_N_ and Ni sites increase the electron transfer of the proximal C‐N bond adsorbed on Ni_3_N‐V_N_, reduce the energy barrier of Ni_3_N‐V_N_ to break the proximal C─N bond, and finally promote the dissociation of the proximal C─N bond.

## 3 Conclusion

In summary, we developed a nitrogen‐vacancy engineering strategy to construct electron‐rich Ni sites and electron‐deficient V_N_ local environments in the Ni_3_N‐V_N_/NF UOR electrocatalyst. This unique microenvironment guides the reaction along the “NCO⁻” pathway and promotes the formation of NiOOH. Due to the dominance of the “NCO⁻” pathway on Ni_3_N‐V_N_/NF, the UOR process undergoes minimal NiOOH/Ni(OH)_2_ conversion during the indirect oxidation reaction, significantly reducing the charge transfer resistance (R_ct_). Furthermore, the electron‐rich Ni sites facilitate the formation of NiOOH, contributing to fast reaction kinetics. The optimized Ni_3_N‐V_N_/NF(M) catalyst demonstrates exceptional UOR performance, achieving a low potential of 1.46 V at 1000 mA cm^−2^, a remarkable Tafel slope of 32.1 mV dec^−1^, and robust stability. When integrated into a practical anion exchange membrane (AEM) electrolyzer, it achieves a current density of 600 mA cm^−2^ at a cell voltage of 1.84 V with high stability, showcasing its potential for industrial applications. The “NCO⁻” pathway minimizes NiOOH/Ni(OH)_2_ conversion because the electron‐deficient V_N_ sites preferentially adsorb the electron‐donating ─NH_2_ group of urea, while the electron‐rich Ni sites promote the dissociation of proximal C─N bonds. Additionally, the electron‐rich Ni sites exhibit d‐band centers closer to the Fermi level, which lowers the energy barrier for NiOOH formation, enabling Ni_3_N‐V_N_ to generate NiOOH at low potentials. This work not only provides a novel strategy for designing UOR catalysts with enhanced activity and stability but also offers new insights into the urea electrooxidation pathway on nickel‐based catalysts. These findings pave the way for the development of efficient and sustainable electrocatalysts for energy conversion applications.

## Conflict of Interest

The authors declare no conflict of interest.

## Supporting information



Supporting Information

## Data Availability

The data that support the findings of this study are available from the corresponding author upon reasonable request.
