# Supplementary material for: Pairing N‐Vacancy and Adjacent Ni‐Sites in the Local Microenvironment to Regulate the Urea Oxidation Reaction Pathway With Enhanced Kinetics
Source: Adv Mater. 2025 Apr 16;37(28):2503879. doi: 10.1002/adma.202503879 (PMC12272033; doi:10.1002/adma.202503879)
Supplement: Supplementary file 1 — Supporting Information [file ADMA-37-2503879-s001.docx]

Supporting Information

Pairing N-Vacancy and Adjacent Ni-Sites in the Local Microenvironment to Regulate the Urea Oxidation Reaction Pathway with Enhanced Kinetics

Chengwei Ji,^#^ Huimei Duan ^#^ Chuanhui Wang, Guizeng Liang, Xiaojing Long, Xilin She, Rongrong Zhang, Feilong Gong, Daohao Li,* Dongjiang Yang,* and Jian Liu,*

**Experimental Section**

**1.** **Materials and Chemicals**

Hydrochloric acid (HCl, 36.0% - 38.0%), ethanol (C_2_H_5_OH, 99.7%), potassium hydroxide (KOH, 85.0%), nickel (ІІ) nitrate hexahydrate (Ni(NO_3_)_2·_6H_2_O, 98.0%), ammonium fluoride (NH_4_F, 96.0%), urea (CH_4_N_2_O, 99.0%), and sodium borohydride (NaBH_4_, 98.0%) were purchased from Sinopharm chemical Reagent Co, Ltd. (China). Platinum on graphitized carbon (Pt/C, 20%) was purchased from Beijing InnoChem Science & Technology Co., Ltd. (China). All chemicals were used without further purification.

**2.** **Synthesis of Ni(OH)_2_/NF**

First, nickel foam was treated with 1 M HCl and cleaned with water. In total, 2.680 g of Ni(NO_3_)_2_·6H_2_O, 2.428 g of urea, and 0.622 g of NH_4_F were dissolved in 160 mL of deionized water. Then, the nickel foam and the above solution were placed in an autoclave at 120 °C for 6 h. After cooling to room temperature, the obtained Ni(OH)_2_ precursor grown on NF (Ni(OH)_2_/NF) was washed several times, and dried in air at 60 ℃ for 2 h.

**3. Synthesis of** **Ni_3_N/NF and Ni_3_N-V_N_/NF**

The Ni(OH)_2_/NF obtained before was annealed at 380 °C for 3 h under NH_3_ atmosphere to obtain the Ni_3_N/NF. To create nitrogen vacancies, Ni_3_N/NF with an area of 6 × 4 cm^2^ was soaked in 20 mL 1.0 _M_ sodium borohydride solution at room temperature for 10, 30, and 50 min to obtain Ni_3_N/NF with low, medium, and high concentration of nitrogen vacancy, respectively, which were named Ni_3_N-V_N_/NF(L), Ni_3_N-V_N_/NF(M), Ni_3_N-V_N_/NF(H). The obtained products were washed several times, and dried in air at 60 ℃ for 2 h. The principle of using sodium borohydride to produce nitrogen vacancy is to use sodium borohydride as a reducing agent to separate the nitrogen part of the lattice to form nitrogen vacancy, the equation is as follows:^[1]^

$\text{2}\text{ }\text{Ni}\text{3}\text{N+2}\text{ }\text{NaBH}\text{4}\text{+4}\text{ }\text{H}\text{2}\text{O}\text{→2}\text{ }\text{Ni}\text{3}\text{N}\text{1-x}\text{+2}\text{ }\text{NaBO}\text{2}\text{+8}\text{ }\text{H}\text{2}\text{+x}\text{ }\text{N}\text{2}$ (1)

**4. Synthesis of Pt/C/NF**

Industrial Pt/C catalyst was dispersed in 100 μL of deionized water, 100 μL of ethanol, and 10 μL of Nafion solution, which was treated with ultrasound for 2 h to form two homogeneous catalyst inks. Catalyst ink was then dropped onto clean NF and dried overnight in room temperature air to obtain NF supported Pt/C electrode, and the loading amount of catalyst is 1 mg/cm^2^.

### **5. Materials characterization**

### The X-ray diffraction (XRD) analysis was carried using a Cu Kα radiation source with a 2θ range of 5 to 90º to identify the phase of the samples. The chemical constitution was investigated by X-ray photoelectron spectroscopy (XPS) using a Thermo Scientific K-Alpha electron spectrometer with Al Kα radiation. Field emission scanning electron microscopy (FESEM) was performed to obtain the morphology and elemental distribution of the as-synthesized samples (JSM-7001F, JEOL, Tokyo, Japan). Scanning transmission electron microscopy (STEM, Thermo Fisher Scientific, G2 80-200) was used to image the morphology and structure of the prepared samples. To prepare the TEM samples, the materials were dispersed in absolute ethanol by ultrasonication for 10 min and then drop–cast onto holey carbon-coated copper TEM grids. Electron paramagnetic resonance (EPR) spectra were recorded by Bruker A300 at room temperature. The X-ray absorption fine structure (XAFS) spectra at Ni K-edge were collected in transmission mode on Table XAFS-500 (Specreation Instruments Co., Ltd.). The samples were ground and tableted into slices with a diameter of 12.7 mm. In-situ FTIR measurements were carried out with zinc selenide crystal as an infrared transmission window using otto-internal reflection mode. The terminal is equipped with an FTIR spectrometer (Nicolet 6700) with an MCT detector (Linglu Instrument Equipment Co., Ltd). The infrared absorption spectrum was scanned 32 times and the resolution was 4 cm^-1^. The in-situ Raman was carried out by a SpectraPro HRS-750 Raman spectrometer (532 nm laser) to track the intermediate evolution during electrocatalysis. For the potential-resolved in-situ Raman spectra, an electrochemical workstation (CHI760E) was used to apply potentials to the working electrodes, and every intended potential was held for 5 min before recording each spectrum to reach steady state conditions. For the time-resolved UOR in-situ Raman spectra, we collected Raman spectra during the chronoamperometric measurements at static potentials in 1.0 M KOH + 0.33 _M_ urea.

### **6. Electrochemical measurements**

### All the electrochemical measurements were performed with a CHI 760E electrochemical test instrument in a standard three-electrode setup. In this work, we used Ag/AgCl as the reference electrode, a platinum wire as the counter electrode, and a catalyst with an area of 1 × 0.2 cm^2^ directly as the working electrode. To evaluate the performance of the as-synthesized catalysts at room temperature, the OER test was performed at 1 _M_ KOH and the UOR test at 1 _M_ KOH and 0.33 _M_ urea. All the potentials were corrected according to the Nernst equation:

E_RHE_ = E_Ag/AgCl_ + 0.197 − 0.059 pH – IR (2)

In this work, linear sweep voltammetry (LSV) was performed at a scan rate of 5 mV·s^−1^ in electrolytes. The electrochemical impedance spectroscopy (EIS) was performed in the frequency range of 100 kHz to 0.01 Hz with the potential changing from 1.25 V to 1.5 V. A constant voltage test was carried out to test the stability of the synthetic catalyst. In addition, to evaluate the intrinsic activity of the as-synthesized catalyst, we calculated the electrochemical active surface area (ECSA) of the as-synthesized catalyst by electrochemical double-layer capacitance (C_dl_). The electrochemical double-layer capacitance (C_dl_) measurements were performed with cyclic voltammetry (CV) curves with different scanning rates of 20, 40, 60, 80, 100, and 120 mV·s^−1^, respectively.

### **7. Electrochemical Cell measurements**

### An anion-exchange membrane (AEM) flow electrolyzer is composed of a titanium plate, catalyst(0.5 × 0.5 cm^2^), and anion exchange membrane. Firstly, Ni_3_N-V_N_/NF(M) and Pt/C/NF are assembled into AEM flow electrolyzer as anode and cathode respectively with anion exchange films (X37-50 grade 60). Then, 1 _M_ KOH and 1 _M_ KOH + 0.33 _M_ urea were injected into the cathode and anode at the same time, and the temperature was 55 ℃. The polarization curves were subsequently measured by linear sweep voltammetry (LSV) at a scan rate of 0.1 V s^-1^.

### **8. Product analysis**

### Determination of nitrate, nitrite, cyanate, and ammonium ion concentration by ion chromatography (America, Thermo Scientific ICS-5000+). Use KOH or mesylate as the eluent. The calibration curve standard is prepared from commercially available anionic or cationic solutions. The reaction mixture was diluted to prepare the IC sample. In addition, carbon-based products (such as cyanates and carbonates) of urea electrolysis were qualitatively analyzed by ^13^C NMR. The nuclear magnetic resonance analysis sample was prepared by mixing 0.8 mL reaction mixture with 0.1 mL deuterium oxide.

### **9. Theoretical calculations**

### The density functional theory (DFT) calculations were carried out using the Vienna ab Initio Simulation package (VASP).^[2]^ The ion-electron interactions were described by the projector plane wave (PAW) approach. Electron exchange–correlations were represented by the functional of Perdew, Burke, and Ernzerhof (PBE) of generalized gradient approximation (GGA).^[3]^ To ensure the convergence for total energy, all calculations were performed using a plane-wave cutoff energy of 500 eV with Monkhorst-Pack grid (2×2×1) used for k-point, the vacuum slab was set to 20 Å, which is large enough to avoid interactions between the slabs. The bottom two layers are fixed to their bulkposition by VASPKIT 1.4.1. Besides, the convergence threshold of energy and forces were set to be 1×10^-4^ eV and -0.05 eV/Å, respectively.

The Gibbs free energy of formation ∆*G* for each step is calculated by the following formula:^[4]^

$\text{∆}\text{G}\text{ }\text{=}\text{ }\text{∆}\text{E}\text{ }\text{+}\text{ }\text{∆}\text{E}_{\text{ZPE}} \text{-}\text{ }\text{T}\text{∆}\text{S}$ (3)

where ∆*E* is the adsorption energy of adsorbed species, and *T* is temperature (*T* was set to be 300 *K*). ∆*E*_ZPE_ and ∆*S* are the energy difference in zero point energy and entropy, respectively.

**Supplementary Figures**


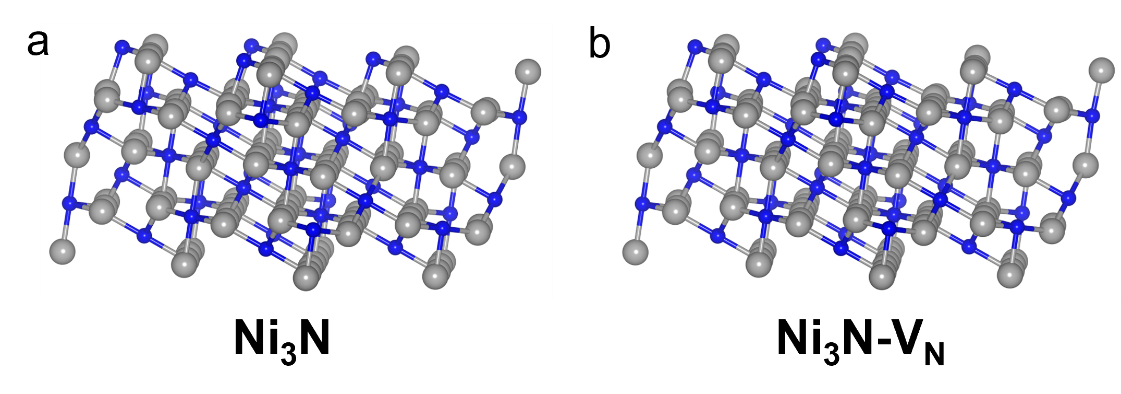


**Figure S1.** The optimized structure models of Ni_3_N (a) and Ni_3_N-V_N_ (b).


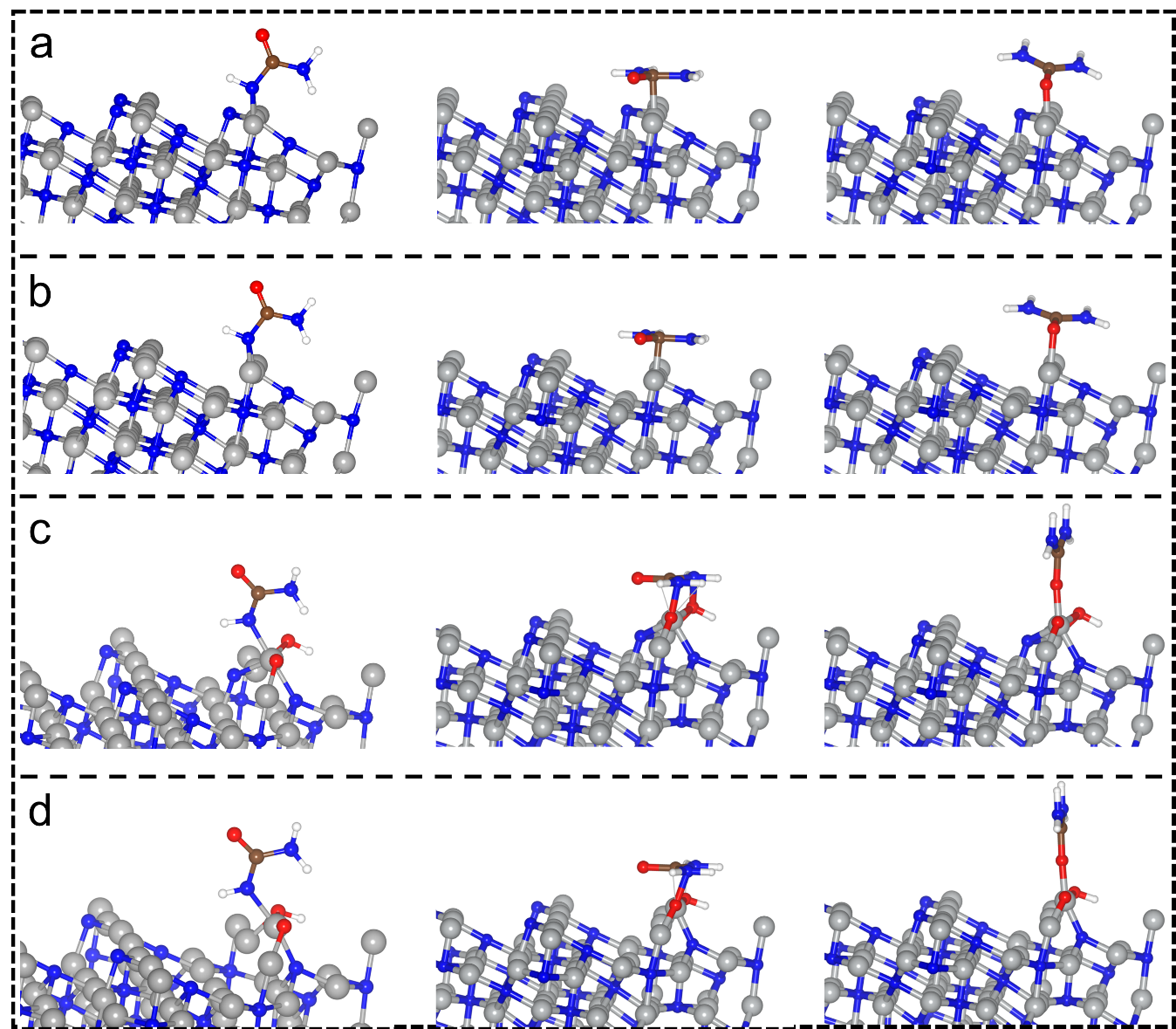


**Figure S2.** The adsorption model of urea on Ni_3_N (a), Ni_3_N-V_N_ (b), Ni_3_N/NiOOH (c) and Ni_3_N-V_N_/NiOOH (d).


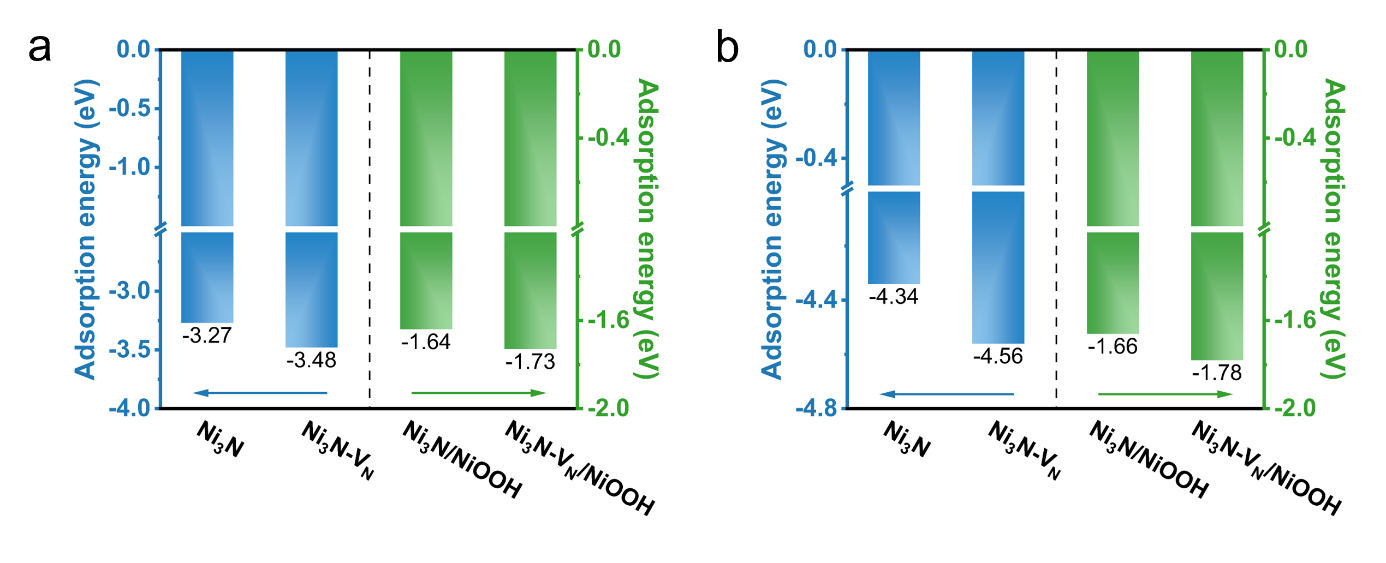


**Figure S3.** The adsorption energy of urea C atom (a) and urea O atom (b) on Ni_3_N, Ni_3_N-V_N_, Ni_3_N/NiOOH and Ni_3_N-V_N_/NiOOH.


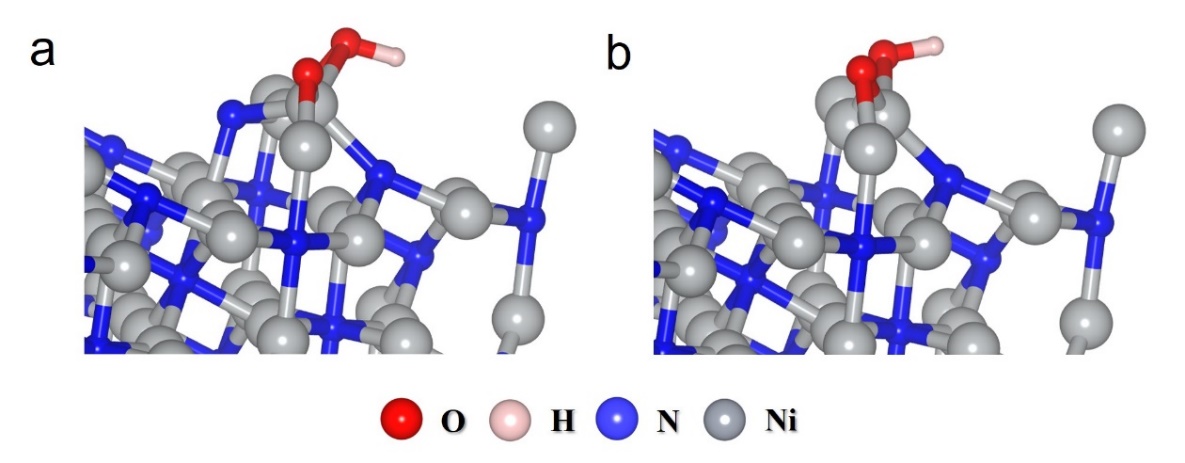


**Figure S4.** The optimized structure models of Ni_3_N/NiOOH (a) and Ni_3_N-V_N_/NiOOH (b).


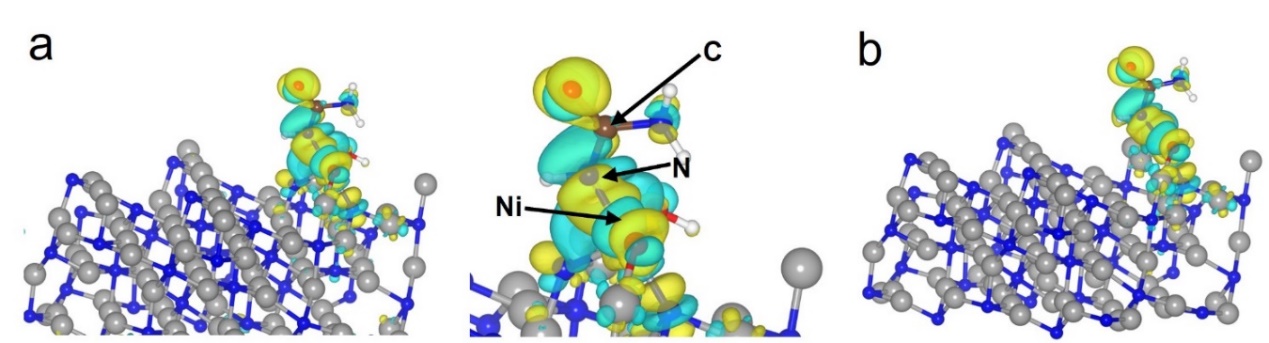


**Figure S5.** The charge density difference of urea molecule on Ni_3_N/NiOOH (a) and Ni_3_N-V_N_/NiOOH (b). The positive and negative charges are shown in yellow and cyan.


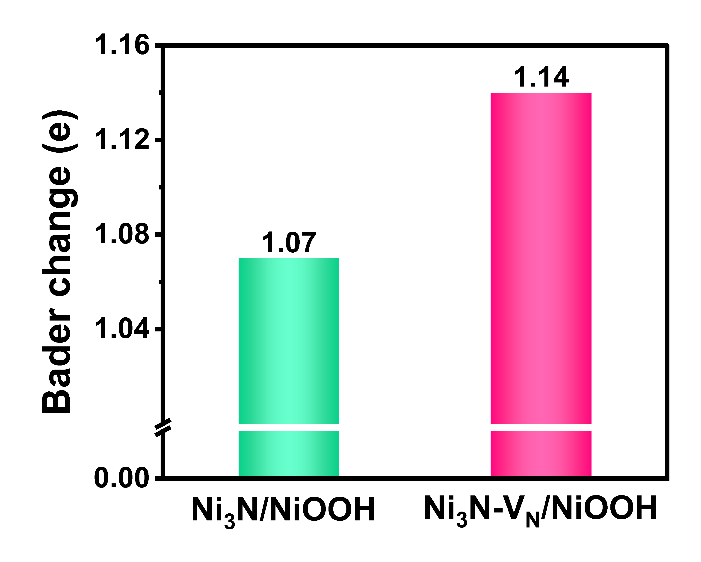


**Figure S6.** The Bader charge analysis diagram of N atom near catalyst on urea.


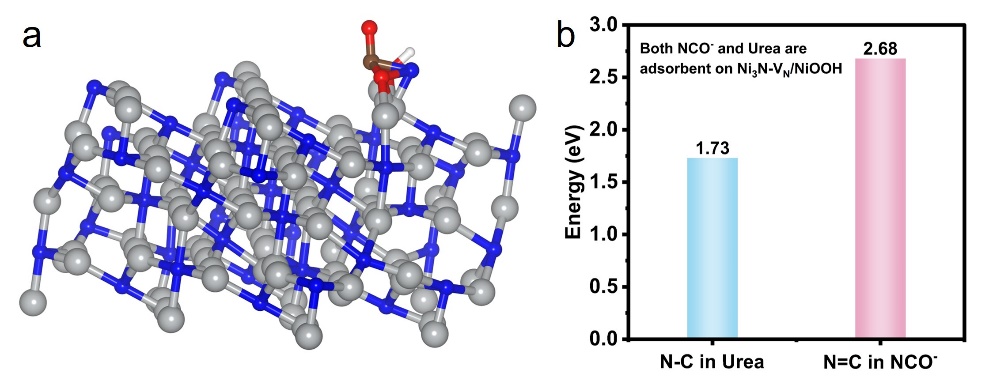


**Figure S7.** a) Model of adsorption of NCO^-^ on Ni_3_N-V_N_/NiOOH. b) Fracture energy of N-C of urea adsorbing on Ni_3_N-V_N_/NiOOH and N=C of NCO^-^ adsorbing on Ni_3_N-V_N_/NiOOH.


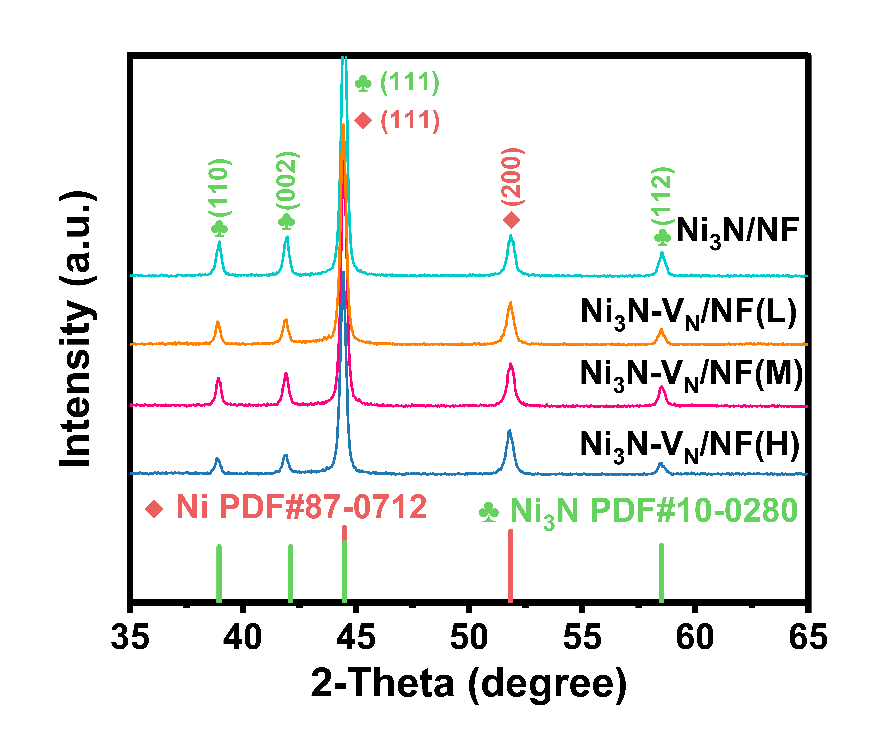


**Figure S8.** XRD pattern of the catalysts treated with different reduction time.


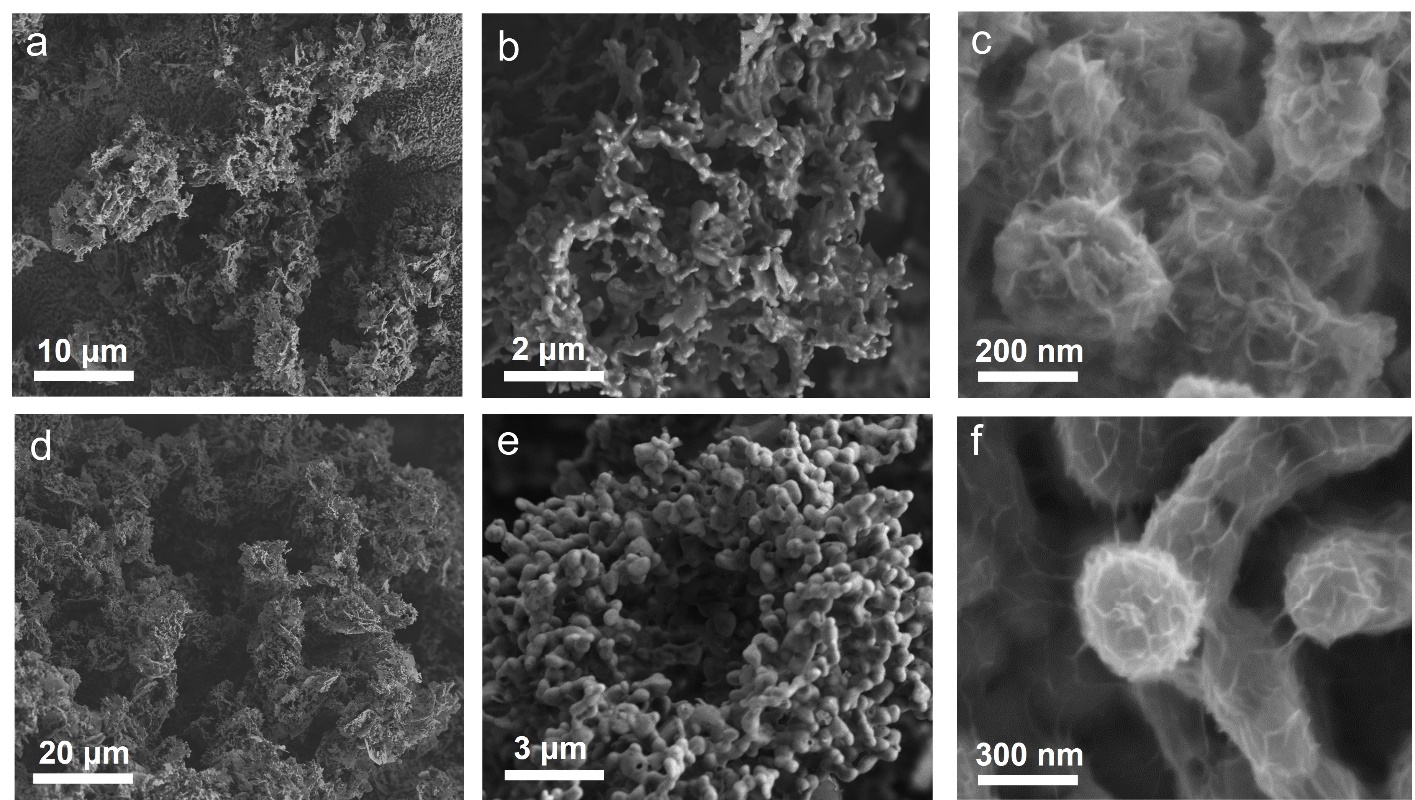


**Figure S9.** SEM images of Ni_3_N/NF (a-c) and Ni_3_N-V_N_/NF(M) (d-f).


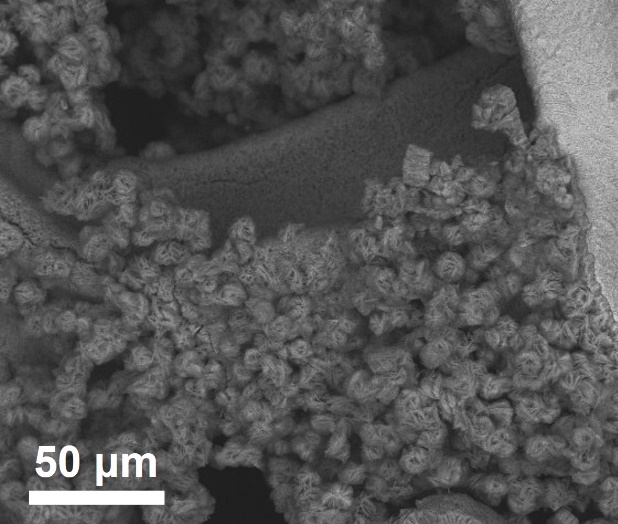


**Figure S10.** SEM images of Ni(OH)_2_.


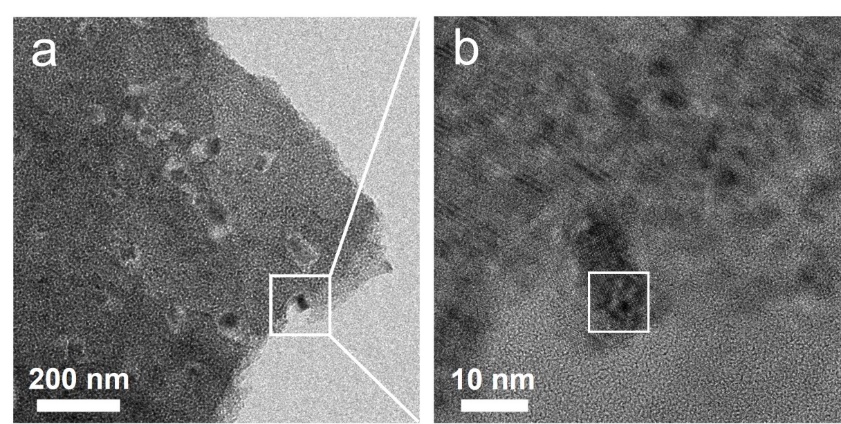


**Figure S11.** TEM (a) and High-resolution TEM (b) images of Ni_3_N-V_N_/NF(M). The white box areas in (a) and (b) correspond to Figure S10a and Figure 2c, respectively.


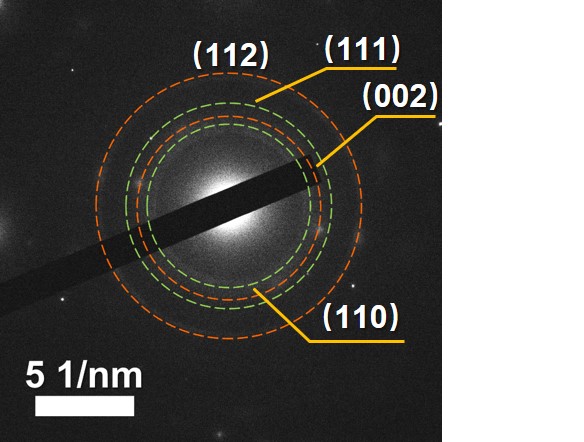


**Figure S12.** SAED images of Ni_3_N-V_N_/NF(M).


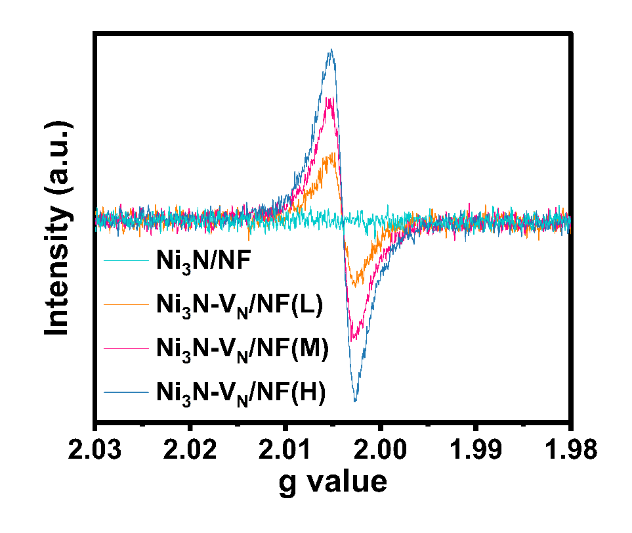


**Figure S13.** EPR spectrum of the catalysts treated with different reduction time.


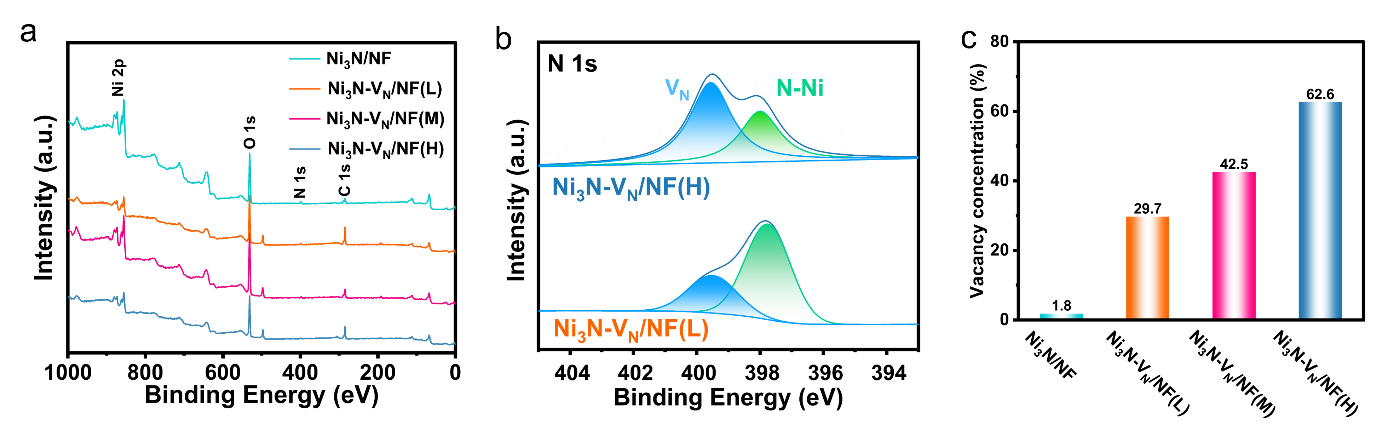


**Figure S14.** a) XPS spectra of survey scan for the prepared catalysts. b) XPS spectra of N 1s for Ni_3_N-V_N_/NF(L), and Ni_3_N-V_N_/NF(H). c) The nitrogen vacancy concentration of the prepared catalysts.


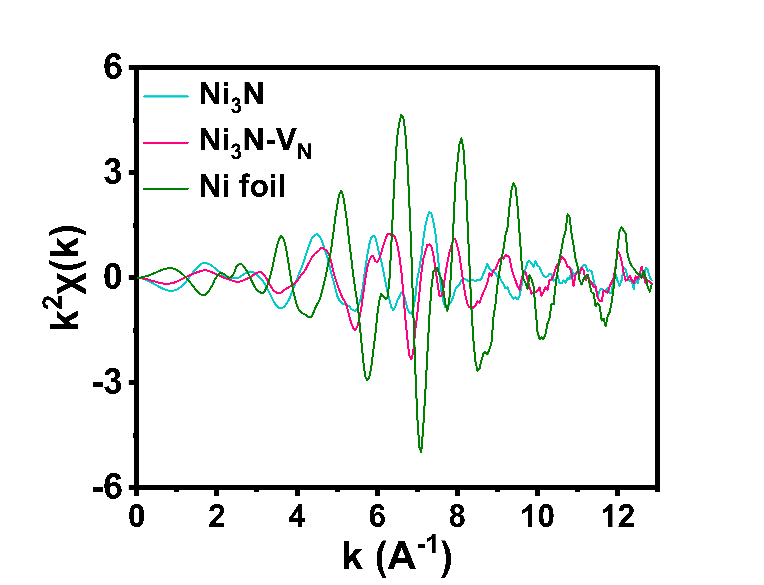


**Figure S15.** K-space EXAFS images of Ni_3_N, Ni_3_N-V_N_, and Ni foil.


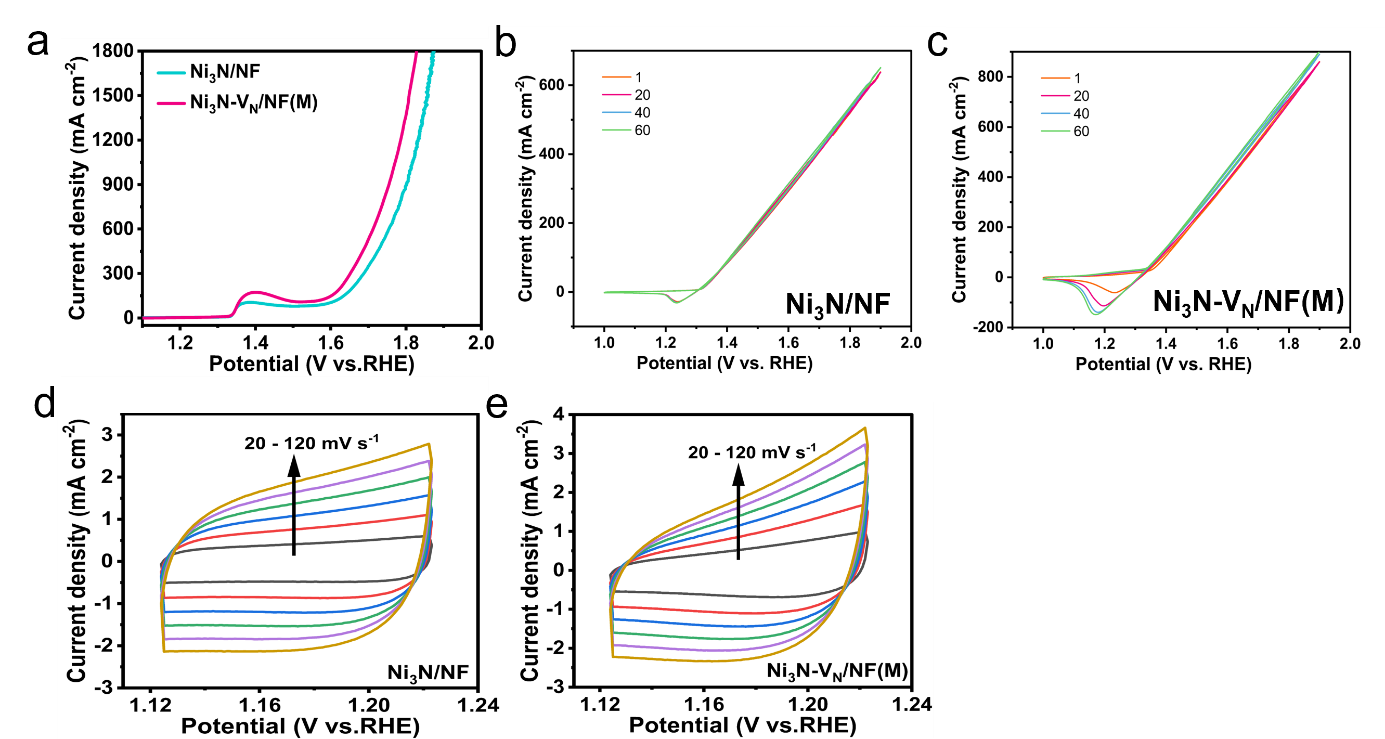


**Figure S16.** a) OER LSV curves of all catalysts in 1.0 _M_ KOH. CV curves of Ni_3_N/NF (b) and Ni_3_N-V_N_/NF(M) (c) for 1, 20, 40, 60 cycles collected in 1 _M_ KOH + 0.33 _M_ urea. CV curves at different scan rate from 20 to 120 mV·s^-1^ of Ni_3_N/NF (d) and Ni_3_N-V_N_/NF(M) (e).

For Figure S16b-c, the phase transition of the catalyst accompanies the entire reaction process of both Ni_3_N-V_N_/NF(M) and Ni_3_N/NF. In the reaction of Ni_3_N-V_N_/NF(M), NiOOH is consumed less and accumulates, leading to an increase in activity after multiple CV cycles. In contrast, in the Ni_3_N/NF reaction, NiOOH is generated and then consumed, and this process continues, so the performance remains stable.


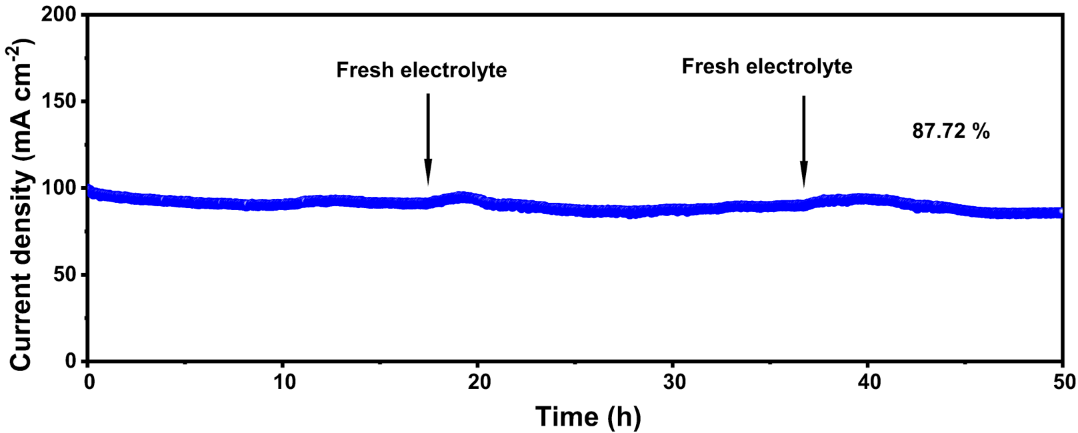


**Figure S17.** Amperometric i-t curves of the Ni_3_N-V_N_/NF(M) in 1.0 _M_ KOH + 0.33 _M_ urea.


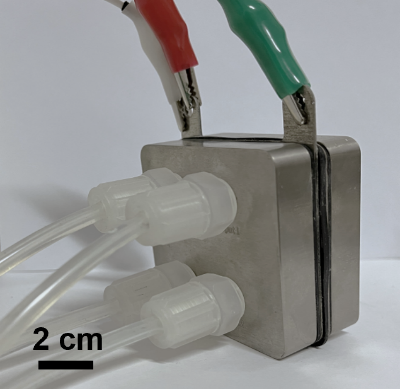


**Figure S18.** A photograph of the AEM cell test unit.


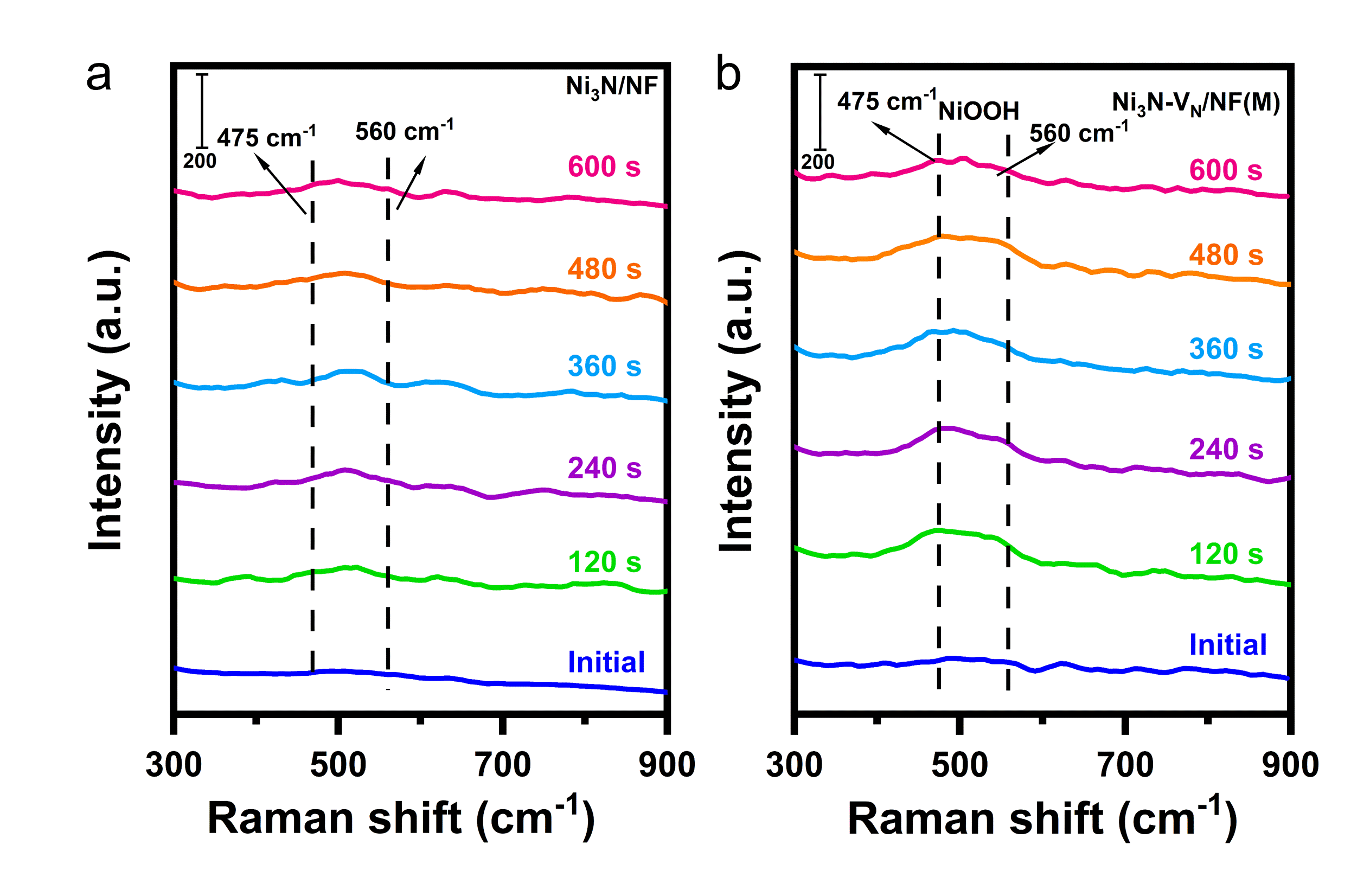


**Figure S19.** The time-resolved in-situ Raman spectra of Ni_3_N/NF (a) and Ni_3_N-V_N_/NF(M) (b) in 1.0 _M_ KOH + 0.33 _M_ urea.

The picture shows the time-resolved in-situ Raman of Ni_3_N-V_N_/NF(M) and Ni_3_N/NF at 1.35 V. Their peak patterns are the same as the 1.35 V curves shown in Fig. 4b and 4a respectively, and there is no significant change from 120 to 600 s. The combination of potential-resolved and time-resolved in-situ Raman spectra show that Ni_3_N-V_N_/NF(M) can be converted to NiOOH at 1.35 V, while Ni_3_N/NF cannot be converted.


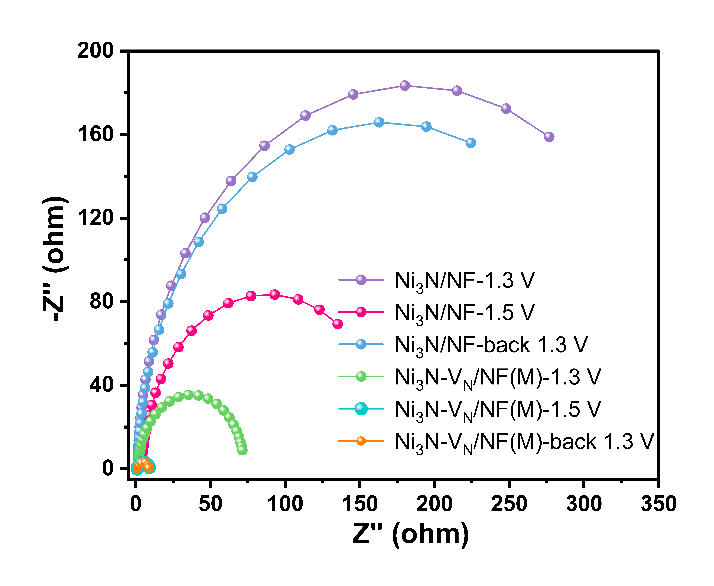


**Figure S20.** EIS Nyquist plot for UOR of Ni_3_N/NF and Ni_3_N-V_N_/NF(M) at different potentials.


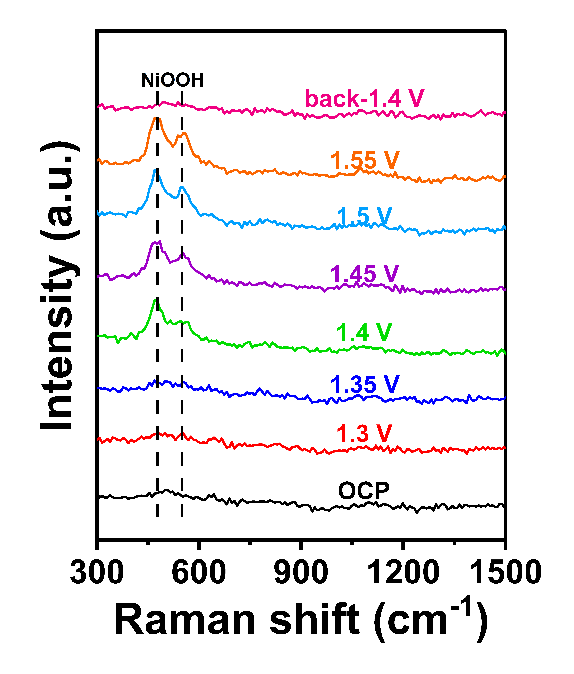


**Figure S21.** The potential-resolved in-situ Raman spectra of Ni_3_N-V_N_/NF(M) in 1.0 _M_ KOH.


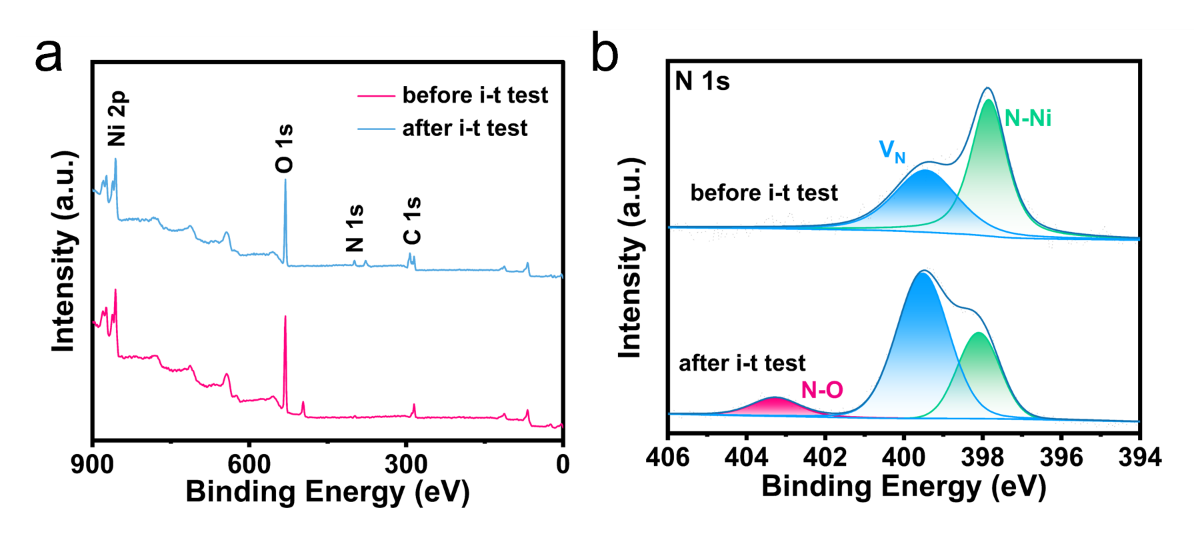


**Figure S22.** XPS spectra of survey scan (a) and N 1s (b) for the initial and recovered Ni_3_N-V_N_/NF(M).


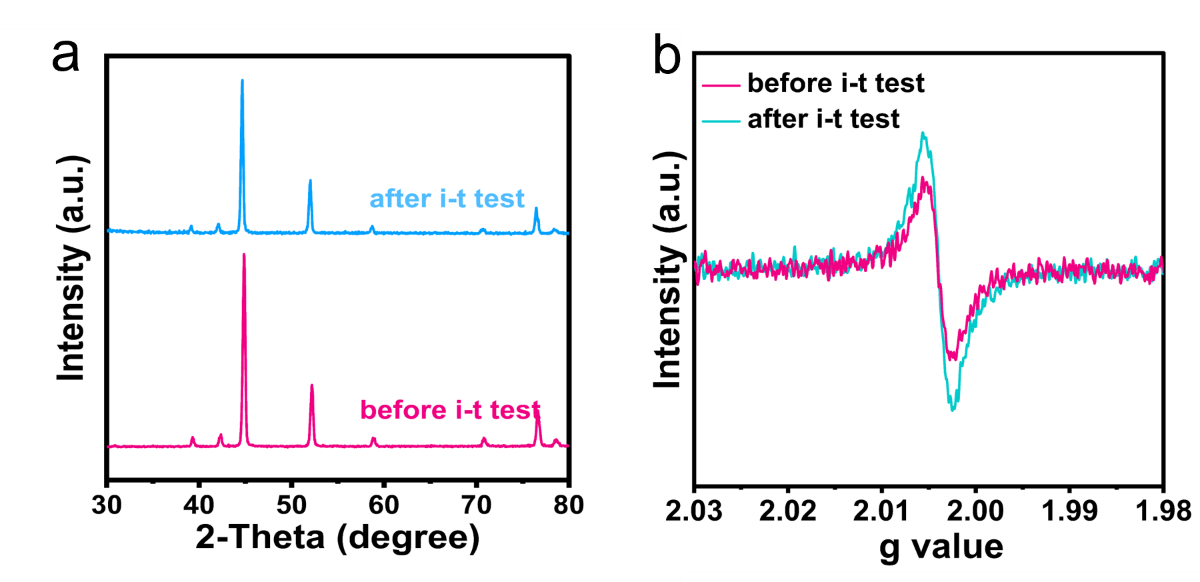


**Figure S23.** XRD pattern (a) and EPR spectrum (b) comparison of the initial and recovered Ni_3_N-V_N_/NF(M).


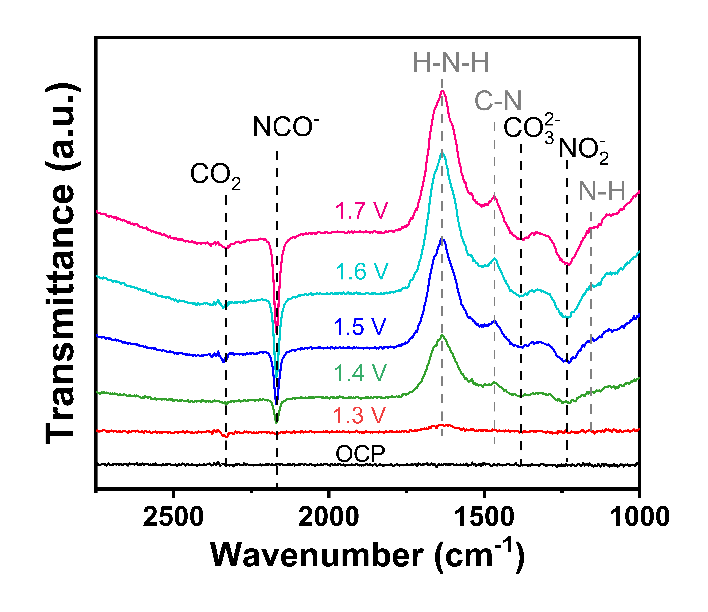


**Figure S24.** In-situ FTIR spectra obtained of Ni_3_N/NF.


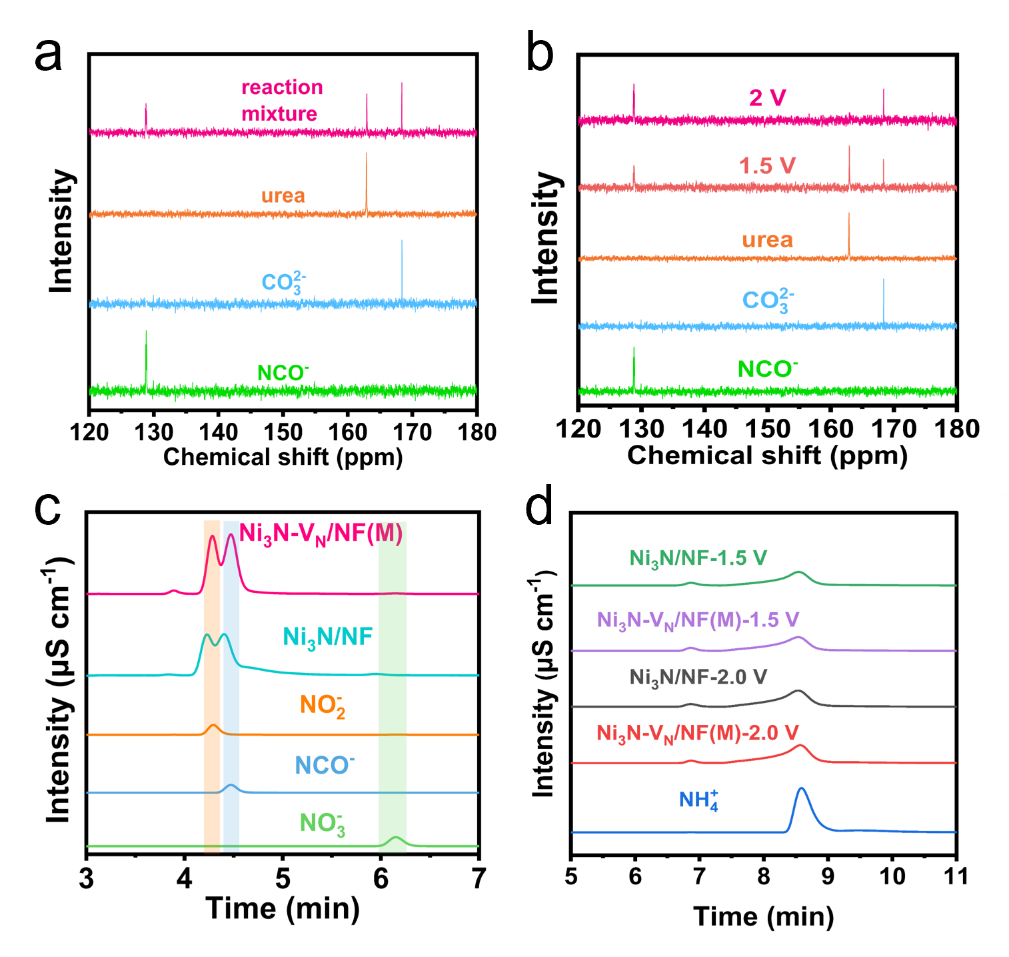


**Figure S25.** Examples of the NMR spectra recorded for long-term UOR at the Ni_3_N-V_N_/NF anode (1.5 V) (a) and Ni_3_N/NF anode (1.5 and 2.0 V) (b) compared to the NMR spectra of several carbon-containing anions and urea. c) Examples of the IC traces recorded for UOR reaction mixture of Ni_3_N/NF and Ni_3_N-VN/NF(M) at 1.5 V compared to several standard anion solutions. d) The IC traces of the UOR reaction mixture of Ni_3_N/NF and Ni_3_N-V_N_/NF(M) were recorded at 1.5 and 2.0 V in comparison with NH_4_^+^ solution. All voltages are relative to RHE, without IR compensation.


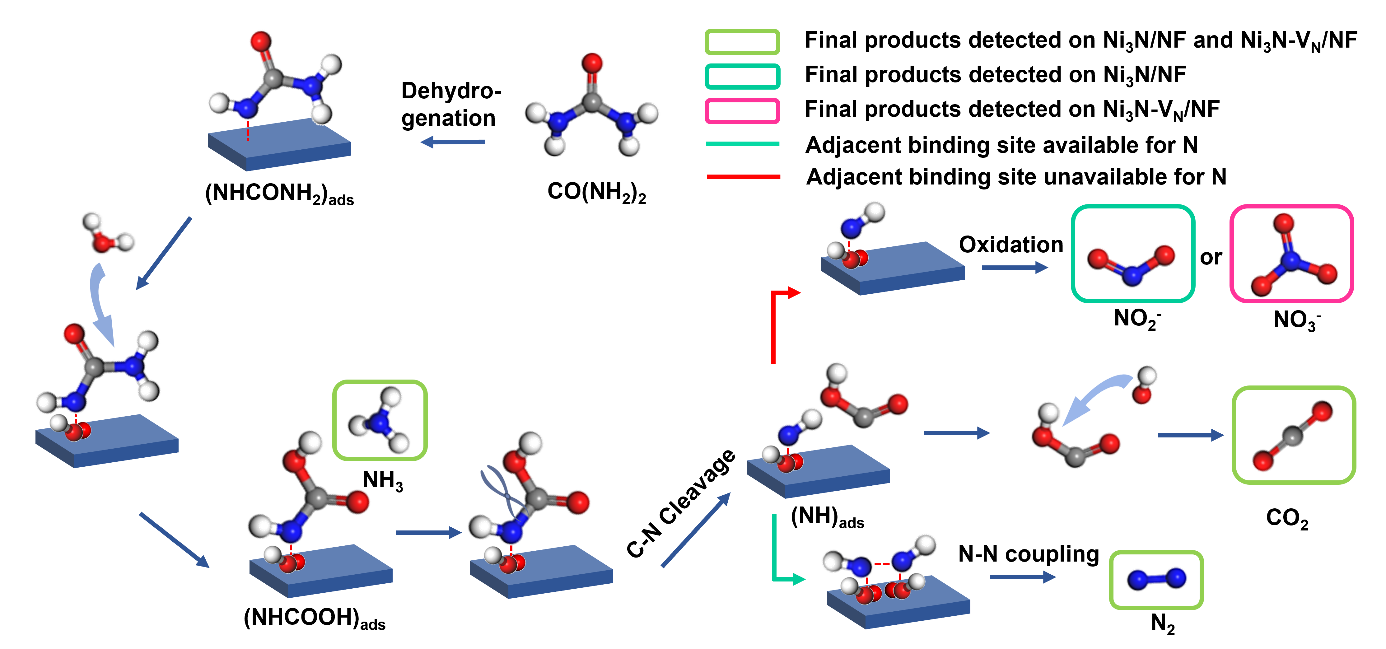


**Figure S26.** A schematic illustration of the proposed pathways for UOR on the catalyst surface.


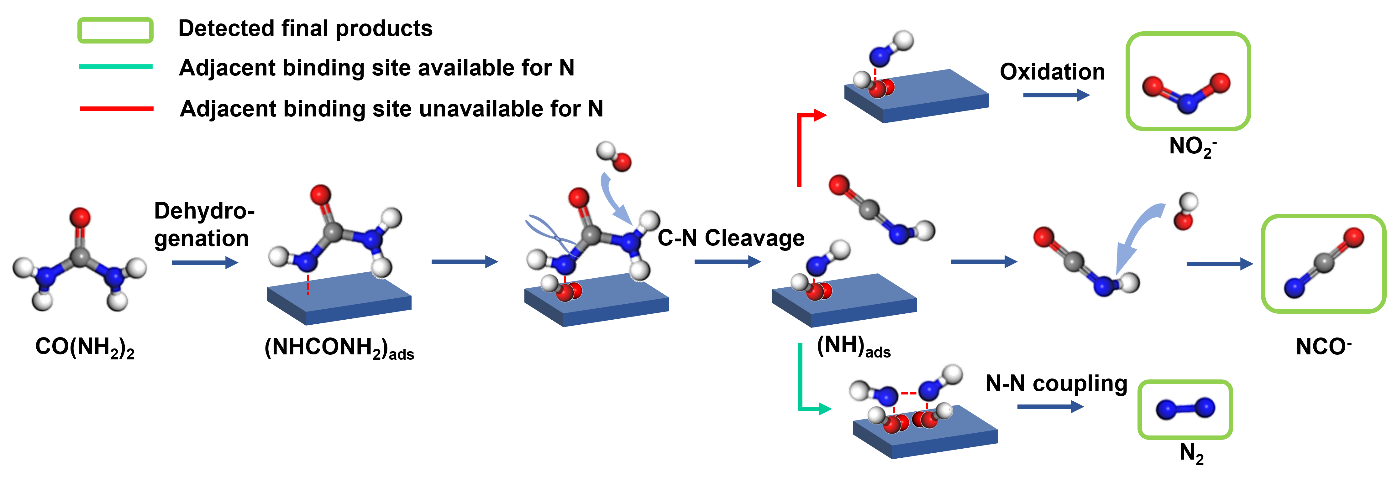


**Figure S27.** A schematic illustration of the proposed pathways for UOR on the Ni_3_N surface.

**Table S1.** Structure parameters of the Ni_3_N and Ni_3_N-V_N_ extracted from the EXAFS fitting of Ni K-edge. (S_0_^2^=0.80)

| Sample | Bond type | CN | R (Å) | σ^2^ (Å^2^) | ΔE_0_ (eV) | R factor |
| --- | --- | --- | --- | --- | --- | --- |
| Ni_3_N | Ni-N | 2.97 | 1.22 | 0.01306 | -6.675 | 0.008 |
|  | Ni-Ni | 5.85 | 2.42 | 0.01274 | -8.005 | 0.008 |
| Ni_3_N-V_N_ | Ni-N | 2.01 | 1.42 | 0.03112 | -1.614 | 0.007 |
|  | Ni-Ni | 4.67 | 2.13 | 0.01153 | -3.405 | 0.007 |

Note: S_0_^2^ is the amplitude reduction factor; CN is coordination number; R is interatomic distance (the bond length between central atoms and surrounding coordination atoms); σ^2^ is Debye-Waller factor (a measure of thermal and static disorder in absorber-scatterer distances); ΔE_0_ is edge-energy shift (the difference between the zero kinetic energy value of the sample and that of the theoretical model). R factor is used to value the goodness of the fitting.

**Table S2.** Comparison of the activities of different Ni_3_N-based UOR electrocatalysts.

| **Catalyst** | **Electrolyte** | **Potential (V vs. RHE)** | **Current density (mA cm^-2^)** | **Catalyst support** | **Reference** |
| --- | --- | --- | --- | --- | --- |
| **Ni_3_N-V_N_/NF(M)** | **1 _M_ KOH+0.33 _M_ Urea** | **1.46/1.33** | **1000/20** | **Nickel foam** | **This work** |
| Ni_3_N/Mo_2_N | 1 _M_ KOH+0.33 _M_ Urea | 1.43 | 400 | Nickel foam | ACS Catal. **2023**, 13, 4091-4100. |
| NiFe(OH)x/Ni_3_N | 1 _M_ KOH+0.33 _M_ Urea | 1.55 | 250 | Nickel foam | ACS Sustainable Chem. Eng. **2021,** 9, 12584–12590. |
| Ni_3_N–400 | 1 _M_ KOH+0.33 _M_ Urea | 1.6 | 70 | Powder | J Electroanal Chem. **2023,** 948, 117821. |
| Ni_3_N/Ni_0.2_Mo_0.8_N/NF | 1 _M_ KOH+0.5 _M_ Urea | 1.328 | 10 | Nickel foam | Chem. Eng. J. **2021**, 409, 128240. |
| C-350 | 1 _M_ KOH+0.5 _M_ Urea | 1.75 | 800 | Nickel foam | ACS Sustainable Chem. Eng. **2020,** 8, 7414–7422. |
| Ni_3_N–C | 1 _M_ KOH+0.5 _M_ Urea | 0.55 (vs Hg/HgO) | 48 | Carbon | ACS Appl. Energy Mater. **2022**, 5, 1397–1402. |
| Ni_3_N–350/NF | 1 _M_ KOH+0.5 _M_ Urea | 1.406 | 100 | Nickel foam | Int J Hydrogen Energ. **2020**, 45, 14199-14207. |
| Ni/VN/NF | 1 _M_ KOH+0.33 _M_ Urea | 1.28 | 10 | Nickel foam | J Alloy Compd. **2023**, 968, 171861. |
| Ni_3_N/rGO@NF-350 | 1 _M_ KOH+0.33 _M_ Urea | 1.342 | 10 | Nickel foam | Nanomaterials. **2019,** 9, 1583. |
| V–Ni_3_N/NF | 1 _M_ KOH+0.5 _M_ Urea | 1.361 | 10 | Nickel foam | J. Mater. Chem. A **2021**, 9, 4159. |

**Table S3.** Comparison of the activities of different AEM electrolyzers.

| **Anode catalyst** | **Cathode catalyst** | **Anode electrolyte** | **Potential (V)** | **Current density (mA cm^-2^)** | **Reference** |
| --- | --- | --- | --- | --- | --- |
| **Ni_3_N-V_N_/NF(M)** | **Pt/C/NF** | **1 _M_ KOH+0.33 _M_ Urea** | **1.84** | **600** | **This work** |
| Ru_1_-Ni(OH)_2_ | P-Ru_1_-Ni(OH)_2_ | 1 _M_ KOH+2 _M_ Urea | 2.0 | 700 | Energy Environ. Sci. **2023**, 16, 6015. |
| Vo_x_-CoP/NF | Vo_x_-CoP/NF | 1 _M_ KOH+0.33 _M_ Urea | 1.77 | 300 | Nano Energy. **2024,** 126, 109613. |
| Mo_2_C/NC@0.5Ni | Mo_2_C/NC@0.5Ni | 1 _M_ KOH+0.33 _M_ Urea | 1.90 | 500 | J ALLOY COMPD. **2023,** 968, 172111. |
| Mo-NiS | Mo-NiS | 1 _M_ KOH+0.5 _M_ Urea | 2.0 | 1000 | Adv. Funct. Mater. **2023**, 33, 2210656. |
| ANH | Pt/C | 1 _M_ KOH+0.33 _M_ Urea | 2.0 | 650 | Adv.Mater. **2023**, 2301549. |
| a-NiCu-NF | a-NiCu-NF | 1 _M_ KOH+0.33 _M_ Urea | 1.60 | 50 | ACS Sustainable Chem. Eng. **2024,** 12, 9908−9921 |
| Ni(OH)_2_/g-C_3_N_4_ | Pt/C/NF | 1 _M_ KOH+0.33 _M_ Urea | 3.0 | 400 | Small **2024**, 2401053, DOI:10.1002/smll.202401053. |
| Ni_2_P-Pt | Ni_2_P-Pt | 1 _M_ KOH+0.33 _M_ Urea | 1.7 | 250 | Angew. Chem. Int. Ed. **2024**, e202407038. |
| CFRO-7 | CFRO-7 | 1 _M_ KOH+0.33 _M_ Urea | 1.54 | 100 | J ENERGY CHEM. **2024**, 92, 233–239. |

Added: All cathode electrolytes are 1 _M_ KOH.

**Table S4.** The concentration of various UOR products was determined by ion chromatography.

| Ion | Ni_3_N/NF  (2.0 V) | Ni_3_N-V_N_/NF(M)  (2.0 V) | Ni_3_N/NF  (1.5 V) | Ni_3_N-V_N_/NF(M)  (1.5 V) |
| --- | --- | --- | --- | --- |
| NO_2_^-^ (mg/L) | 2931.5958 | 290.4639 | 2295.6606 | 6499.271 |
| NO_3_^-^ (mg/L) | 987.9667 | 8305.5196 | 270.1128 | 209.0294 |
| NH_4_^+^ (mg/L) | 4897.2255 | 5766.3624 | 3663.1313 | 4149.7211 |
| NCO^-^ (mg/L) | 6903.8842 | 14425.6891 | 6128.2931 | 12726.5424 |

In the ion chromatography test, the two characteristic peaks of NCO^-^ and NO_2_^-^ partially overlap due to technical problems, so the concentration of NCO^-^ and NO_2_^-^ cannot be accurately quantified, but the change of the concentration ratio of NCO^-^ and NH_4_^+^ in Ni_3_N/NF and Ni_3_N-V_N_/NF(M) can still be predicted based on the test results. First, the NH_4_^+^ content can be accurately determined by ion chromatography (see Supplementary Table 4), and the NH_4_^+^ concentration in Ni_3_N/NF and Ni_3_N-V_N_/NF(M) UOR products does not change much. Secondly, in the ion chromatography test, the sample concentration is not only related to the peak area, but also to the peak height. In the concentration gradient detection of NCO^-^ standard material, we found that the higher the concentration, the higher the peak. Compared with the peak height of NCO^-^ product of Ni_3_N/NF(M), the peak height of NCO^-^ product of Ni_3_N-V_N_/NF(M) increased significantly, indicating that the concentration of NCO^-^ product of Ni_3_N-V_N_/NF(M) increased significantly. In the case of little change in NH_4_^+^ concentration, this also indicates that there are more NCO^-^ than NH_4_^+^ in the UOR products of Ni_3_N-V_N_/NF(M), Ni_3_N-V_N_/NF(M) is more inclined to the "NCO^-^" path.

| Ion | Peak height |
| --- | --- |
| NCO^-^ standard material 1ppm | 0.921 |
| NCO^-^ standard material 5ppm | 4.556 |
| NCO^-^ standard material 10ppm | 9.846 |
| NCO^-^ standard material 20ppm | 16.941 |
| NCO^-^ in products of Ni_3_N/NF (diluted 1000 times) | 3.602 |
| NCO^-^ in products of Ni_3_N-V_N_/NF(M) (diluted 1000 times) | 12.757 |

**Notes S1. Cost calculation of H_2_ production in AEM electrolyzer**

These calculations only considered the electricity costs, based on the method proposed by literature.^[5]^

***H_2_ production rate @ 0.5 A cm^-2^***

= (j A cm^-2^) (1 e^-^/1.602 ×10^-19^ C) (1 H_2_/2 e^-^)

= 0.5 A cm^-2^ / (1.602 × 10^-19^ C × 2)

= 2.59 × 10^-6^ mol H_2_ cm^-2^ s^-1^

***Lower Heating Value (LHV) of H_2_***

= 120 kJ g^-1^ H_2_= 2.42 × 10^5^ J mol^-1^ H_2_

***H_2_ power out***

= (2.59 × 10^-6^ mol cm^-2^ s^-1^) × (2.42 × 10^5^ J mol^-1^)

= 0.627 W cm^-2^

***Electrolyzer Power @ 0.5 A cm^-2^***

= (0.5 A cm^-2^) (1.797 V)

= 0.899 W cm^-2^

***Price per gasoline-gallon equivalent (GGE) H_2_***

= 1GGE H_2_/H_2_ production rate × Electrolyzer power × Electricity bill

= 0.997 kg / (2.59 × 10^-6^ mol H_2_ cm^-2^ s^-1^ × 2 kg/mol) × 0.899 W cm^-2^ × $ 0.02/kW h

= $ 0.96/GGE H_2_

<< $ 2/GGE H_2_ of U.S. Department of Energy (DOE) by 2026.^[6]^

**References**

[1] K. Zhu, F. Shi, X. Zhu, W. Yang, *Nano Energy.* **2020**, *73*, 104761.

[2] G. Kresse, J. Furthmüller, *Phys. Rev. B.* **1996**, *54*, 11169.

[3] J. P. Perdew, K. Burke, M. Ernzerhof, *Phys. Rev. Lett.* **1996**, *77*, 3865.

[4] J. K. Nørskov, J. Rossmeisl, A. Logadottir, L. Lindqvist, J.R. Kitchin, T. Bligaard, H. Jonsson, *J. Phys. Chem. B.* **2004**, *108*, 17886.

[5] X. Kang, F. Yang, Z. Zhang, H. Liu, S. Ge, S. Hu, S. Li, Y. Luo, Q. Yu, Z. Liu, Q. Wang, W. Ren, C. Sun, H-M. Cheng, B. Liu, *Nat. Commun.* **2023**, *14*, 3607.

[6] U.S. Department of Energy. DOE National Clean Hydrogen Strategy and Roadmap. https://www.hydrogen.energy.gov/pdfs/cleanhydrogen-strategy-roadmap.pdf (**2022**).
